# Ecological dynamics and microbial community composition of *Heliotropium arboreum* in the coastal ecosystems of Hainan province

**DOI:** 10.3389/fmicb.2025.1611262

**Published:** 2025-07-03

**Authors:** Xiaofeng Zhang, Fazhi Fang, Linhua Sha, Mir Muhammad Nizamani

**Affiliations:** ^1^Hainan Academy of Forestry (Hainan Academy of Mangrove), Haikou, China; ^2^Key Laboratory of Tropical Forestry Resources Monitoring and Application of Hainan Province, Haikou, China; ^3^Innovation Platform for Academicians of Hainan Province, Haikou, China; ^4^Institute of Marine Sciences, Guangdong Provincial Key Laboratory of Marine Disaster Prediction and Prevention, Shantou University, Shantou, China

**Keywords:** microbial diversity, bacterial communities, fungal communities, environmental factors, South China Sea

## Abstract

*Heliotropium arboreum*, a resilient plant species found in the coastal ecosystems of Hainan Province, thrives in nutrient-poor, isolated habitats. This study examines the microbial community composition and ecological dynamics of *H. arboreum* across several islands in the South China Sea, aiming to understand how environmental factors like soil pH, nitrogen (N), and phosphorus (P) availability influence the plant's microbial communities and ecological success. The study was conducted across seven plots located on different islands, including Zhaoshu Island, Yongxing Island, and Wuzhizhou Island, among others. Soil samples were collected from three layers: 0–10 cm, 10–20 cm, and 20–30 cm. A total of 63 soil samples were processed for physicochemical analysis, measuring pH, organic matter, total nitrogen (TN), total phosphorus (TP), available phosphorus (AP), available potassium (AK), ammonium nitrogen (${\text{NH}}_{4}^{+}$), and nitrate nitrogen (${\text{NO}}_{3}^{-}$). Microbial DNA was extracted, amplified, and sequenced using Illumina-based platforms, producing 5,272,525 high-quality bacterial sequences and 5,273,089 fungal sequences. Bacterial communities were primarily composed of Proteobacteria (range: 29–50%), Planctomycetota, and Acidobacteriota, with significant correlations between certain bacterial genera and nutrient availability. Notably, *Bryobacter* (*r* = 0.810^***^) and *Stenotrophobacter* (*r* = 0.496^***^) showed strong positive correlations with nitrogen availability. Fungal communities were dominated by Ascomycota, followed by Basidiomycota. *Preussia* (*r* = 0.585^***^) and *Metacordyceps* (*r* = 0.616^***^) were positively correlated with nutrient availability. Diversity indices indicated that bacterial diversity was highest in pristine environments such as Zhaoshu Island (350 species), while fungal diversity was lower, with a maximum of 130 fungal species in less disturbed plots. The findings suggest that nutrient availability, particularly nitrogen and phosphorus, significantly shapes the microbial communities associated with *H. arboreum*. Bacterial diversity was positively correlated with nutrient-rich environments, while fungal diversity, especially Ascomycota, was more sensitive to disturbances, particularly in areas like Wuzhizhou Island and Yongxing Island, where human activity is higher. These results highlight the importance of maintaining nutrient availability and reducing human interference to preserve microbial diversity and the ecological resilience of *H. arboreum* in coastal ecosystems.

## 1 Introduction

Coastal and island ecosystems, characterized by saline soils, nutrient limitations, and exposure to harsh climatic conditions such as typhoons and sea-level rise, present formidable challenges for plant survival (Grech et al., 2016; Hernández-Delgado, 2024). These environments often act as natural laboratories for studying plant adaptation due to their fragmented landscapes and extreme abiotic stressors (Warren et al., 2015). Among the resilient species thriving in these settings is *H. arboreum*, a plant endemic to the tropical island coasts, renowned for its ability to colonize isolated, nutrient-poor islands (Zhang et al., 2024; Cai et al., 2020). Its ecological success in such oligotrophic habitats may be mediated by symbiotic interactions with soil microbial communities, which enhance host resilience through nutrient acquisition, stress hormone modulation, and organic matter decomposition (Hassani et al., 2018; Compant et al., 2019; Van Der Heijden et al., 2008). For instance, rhizobia and mycorrhizal fungi are known to mitigate phosphorus limitation in coastal soils a common constraint for island flora (Richardson et al., 2009; Smith and Read, 2008). However, the composition, diversity, and environmental drivers of microbial communities associated with *H. arboreum* across geographically fragmented habitats remain poorly explored, limiting our understanding of its adaptive strategies and the stability of these ecosystems under climate change (Cavicchioli et al., 2019).

Plant-associated microbial communities are profoundly influenced by abiotic factors such as soil pH, nutrient availability, and geographical isolation, which collectively shape microbial structure and function (Fierer and Jackson, 2006; Tedersoo et al., 2014; Bahram et al., 2018). While prior studies have established links between soil chemistry and microbial diversity in terrestrial ecosystems (Bardgett and Van Der Putten, 2014; Delgado-Baquerizo et al., 2018), island environments exhibit unique dynamics due to dispersal barriers, stochastic colonization events, and localized environmental gradients (Weiher et al., 2011; Whitaker et al., 2017). For example, MacArthur and Wilson (2001) theory of island biogeography posits that species richness is governed by island size and isolation, a framework increasingly applied to microbial communities (Nemergut et al., 2013; Nuñez et al., 2009). Yet, the interplay between nutrient constraints (e.g., nitrogen and phosphorus) and microbial community assembly in coastal island systems remains unresolved, particularly in the context of plant-microbe feedback under salinity stress (Egamberdieva et al., 2019; Serna-Chavez et al., 2013).

Understanding the microbial landscape around *H. arboreum* is crucial for elucidating its ecological dynamics and informing conservation strategies in these biodiversity hotspots, which face escalating threats from anthropogenic activities and climate change (Bellard et al., 2014; Corlett, 2020). Given the environmental heterogeneity across islands, it is essential to investigate how nutrient availability, soil pH, and spatial isolation influence microbial diversity and function. Such insights could reveal keystone taxa or functional guilds critical for *H. arboreum* survival, such as nitrogen-fixing bacteria in nitrogen-depleted soils (Reed et al., 2011) or phosphate-solubilizing fungi in highly weathered substrates (Alori et al., 2017). Furthermore, identifying microbial indicators of ecosystem health could enhance restoration efforts in degraded coastal habitats (Wubs et al., 2016; Wagg et al., 2014).

The primary objective of this study is to explore the microbial community composition and diversity associated with *H. arboreum* across multiple islands in the South China Sea and Hainan Province. By integrating geochemical analyses, microbial metabarcoding, and spatial modeling, we examine the influence of soil pH, nitrogen and phosphorus availability, and geographical isolation on microbial assembly. Additionally, we investigate how microbial functional potential correlates with the ecological success of *H. arboreum*, offering insights into the plant's adaptation to harsh island environments and the ecological resilience of these vulnerable ecosystems. This work bridges critical gaps in island biogeography theory and coastal plant-microbe interactions, with implications for biodiversity conservation and ecosystem management in the Anthropocene (Pecl et al., 2017). This study hypothesizes that environmental factors such as soil pH, nutrient availability (specifically nitrogen and phosphorus), and geographical isolation significantly shape the microbial community composition and diversity associated with *Heliotropium arboreum* across coastal islands in the South China Sea and Hainan Province. It is further hypothesized that higher microbial diversity, particularly bacterial communities, correlates with the ecological success and resilience of *H. arboreum*, offering key insights into how plant-microbe interactions contribute to the plant's ability to thrive in nutrient-poor and isolated environments.

## 2 Materials and methods

### 2.1 Information and distribution of sample plots

**Figure 1:**
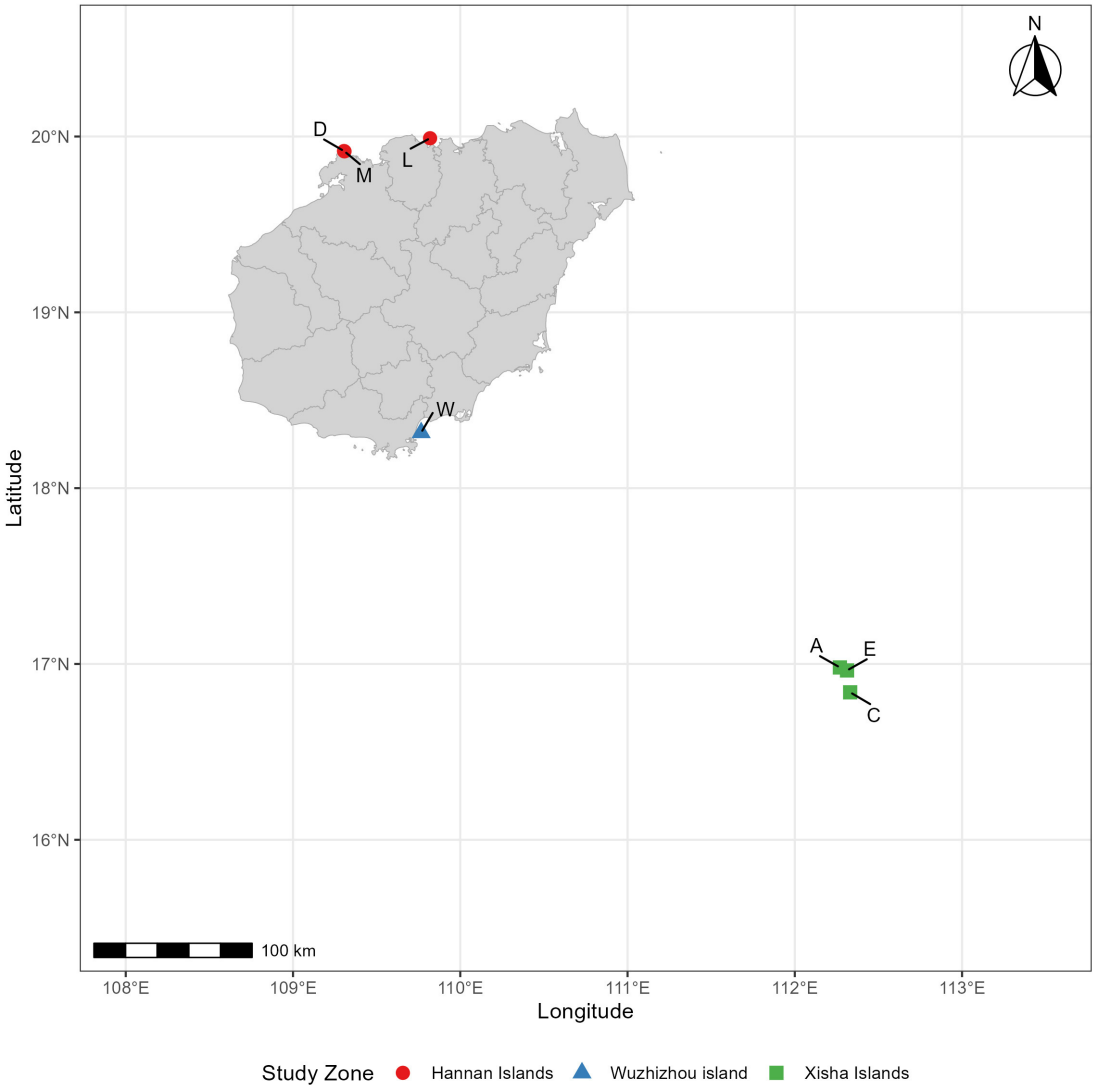
Study area map of the south China sea region showing research sites in the Xisha islands, Wuzhizhou island, and Hannan islands with population abbreviations (C, A, E, W, L, D, E).

This study includes seven plots, each representing a distinct soil type and location (Table 1; Figure 1). Plot A represents Zhaoshu Island, while the designations Au, Am, and Al correspond to the topsoil (post-debris removal 0–10 cm), middle soil (10–20 cm), and subsoil (20 cm−30 cm) of Plot A, respectively. The names u, m, and l refer to the upper, middle, and lower soil layers, respectively. Plots Zhaoshu Island (A), Yongxing Island (C), and North Island (E) are located in Sansha City, Hainan Province, part of the Xisha Islands in the South China Sea. These isolated islands are distant from the mainland and feature a harsh environment, primarily composed of coral sand. Plot L is located in the coastal zone of Lingao County, Hainan Island, and is characterized by coral reefs and rocks. Plot D and plot M are located in the same environmental zone, along the tourist highway in Eman Coast, Danzhou City. The survey sampling line crosses the two places and samples are collected at two points. Although the environmental conditions of these three plots are similar, plots D and M are located near tourist roads, so compared with other plots, human activities and interference are greater. Plot wuzhizhou Island (W) is located on an independent island near Hainan Island, which has notable tourism development, with coral sand and reefs as its dominant features. After further investigation, it was found that, apart from undeveloped islands in the South China Sea, the *H. arboreum* currently exists only in these regions on Hainan Island. Associated species such as Scaevola taccada, Opuntia dillenii, Pandanus urophyllus, and others are also present. However, due to the impact of human development on these islands, much of the coastal vegetation has been cleared. Cultivated garden plants, such as Cocos nucifera, Terminalia catappa, and Morinda citrifolia, have been introduced to the area. Notably, the *H. arboreum* in Plot W and Plot C, which are exposed to substantial human activity, have grown quite tall. Despite the unique environment and the characteristics of *H. arboreum*, no seedling specimens have been found in these locations.

**Table 1:** Detailed information of the research site and population abbreviations.

**Zone**	**Name of research site**	**Population abbreviations**	**Longitude**	**Latitude**	**Other species of transect**
Xisha Islands	Yingxing island	C	112°19′51.07″	16°50′19.67″	*Scaevola taccada, Morinda citrifolia, Cocos nucifera, Pisonia grandis, Terminalia catappa, Casuarina equisetifolia, Codiaeum variegatum*
	Zhaoshu island	A	112°16′10.51″	16°58′48.91″	*Scaevola taccada, Cocos nucifera, Pisonia grandis, Pemphis acidula, Portulaca oleracea*
	Bei island	E	112°18′44.21″	16°57′47.46″	*Scaevola taccada, Cocos nucifera, Pandanus furcatus*
Wuzhizhou island	Wuzhizhou island	W	109°45′57.99″	18°18′56.96″	*Scaevola taccada, Cocos nucifera, Lannea coromandelica, Volkameria inermis, Pongamia pinnata, Pandanus furcatus, Dracaena draco*
Hannan Islands	Lingao coastal	L	109°49′9.64″	19°59′24.66″	*Scaevola taccada, Pandanus furcatus, Opuntia dillenii, Casuarina equisetifolia, Hibiscus tiliaceus*
	Danzhou coastal	D	109°18′17.13″	19°55′2.11″	*Scaevola taccada, Cocos nucifera, Opuntia dillenii, Pandanus furcatus, Terminalia catappa*
		M	109°18′27.43″,	19°54′56.37″	*Scaevola taccada, Opuntia dille, Terminalia catappa*

### 2.2 Soil sampling and physicochemical properties

Soil investigation and sampling were conducted from March to May 2024. A 10 m × 10 m quadrat was established for each plot, with three parallel quadrats set up per plot. The five-point sampling method was employed to collect samples from each quadrat. After removing surface impurities, soil samples were taken from three distinct soil layers: the upper layer (0–10 cm), middle layer (10–20 cm), and lower layer (20–30 cm), using a round knife. For each sample, the soil from five sampling points within the same quadrat and soil layer was mixed. The samples were then placed into sterile self-sealing bags, labeled with the corresponding sample number and collection date, and grouped according to the sample number and soil layer. The collected soil samples were stored at a low temperature for preservation. In the laboratory, the samples were further processed by removing any remaining impurities. After screening through 2 mm micropores, the soil was divided into two portions: 5 g of the sample was placed in a sterile centrifuge tube and sent to the company for sequencing, while another 10 g of soil was naturally dried and stored in sterile self-sealing bags for analysis of the soil's physical and chemical properties. All treatments were completed within 24 h, and a total of 63 soil samples were collected. The physicochemical properties of the soil, including pH value, organic matter, total nitrogen (TN), total phosphorus (TP), total potassium (TK), ammonium nitrogen (${\text{NH}}_{4}^{+}$), available phosphorus (AP), available potassium (AK), and nitrate nitrogen (NO_3_^−^), were measured using methods previously reported (Supplementary Table 1) (Fang et al., 2023).

### 2.3 DNA extraction, PCR amplification and illumina-based sequencing

Total DNA from the soil samples was extracted using the PowerSoil DNA Isolation Kit (MO BIO Laboratories, San Diego, CA, USA) following the manufacturer's instructions. The integrity and purity of the extracted DNA were assessed via 1% agarose gel electrophoresis, and the concentration and purity were measured using a NanoDropOne spectrophotometer. The DNA extracts were then combined for subsequent PCR amplification and sequencing. Soil fungal DNA was amplified using full-length primers: ITS5-1737F (5′-GGAAGTAAAAGTCGTAACAAGG-3′) and ITS2-2043R (5′-GCTGCGTTCTTCATCGATGC-3′). For bacterial DNA, primers 515F (5′-barcode-GTGCCAGCMGCCGCGG-3′) and 907R (5′-CCGTCAATTCMTTTRAGTTT-3′) were used to amplify the V4-V5 regions of the bacterial 16S rRNA gene (Qiu et al., 2021). PCR amplification was performed with the following conditions: initial denaturation at 95°C for 5 min, followed by 27 cycles of 30 s at 95°C, 30 s of annealing at 55°C, and extension at 72°C for 60 s, with a final extension at 72°C for 10 min. The PCR products were purified, quantified, and homogenized to create a sequencing library. After constructing the library and checking its quality, the qualified library was sequenced using the PacBio platform. Guangdong Meige Gene Technology Co., Ltd. carried out the DNA extraction, PCR amplification, and sequencing. To process the raw sequencing data, the quality of the original fastq files was filtered using Trimmomatic. After quality filtering, the valid clean tags were denoised using the unoise3 algorithm with default settings. Chimera removal was performed, and the sequences were merged with FLASH, as previously described (Liu et al., 2020).

### 2.4 Bioinformatics and statistical analysis

Sequences were processed using USEARCH (version 10.0) for row clustering based on a 97% similarity threshold (Quast et al., 2012). Operational Taxonomic Units (OTUs) were filtered with a threshold of 0.005% of all sequenced reads (Kõljalg et al., 2013). Species annotation of OTU sequences was performed using the Unite database (Release 8.0; https://unite.ut.ee/) and the Silva database (Release 132; http://www.arb-Silva.de), with a similarity threshold of 0.8 (Kõljalg et al., 2005; Callahan et al., 2016). For functional ecological guild analysis, FUNGuild (Nguyen et al., 2016) was used to classify fungal taxa by their ecological function. Additionally, BugBase (Ward et al., 2017) was employed to predict bacterial phenotypes.

Statistical analysis was performed using R software. We calculated diversity indices, including Chao1, Shannon, Simpson, and ACE, using both the USEARCH -alpha_div tool (V10, http://www.drive5.com/usearch/) and the R package. Community composition and species abundance were assessed, and the statistical significance of group differences was tested using one-way ANOVA with FDR correction. The Bray-Curtis distance algorithm, implemented in the vegan package of R, was used to conduct Non-metric Multidimensional Scaling (NMDS) analysis. The correlation between community structure and environmental factors was analyzed based on OTU abundance and environmental data, and the results were visualized using a heatmap.

## 3 Results

### 3.1 Distribution and diversity of microbial taxa

**Figure 2:**
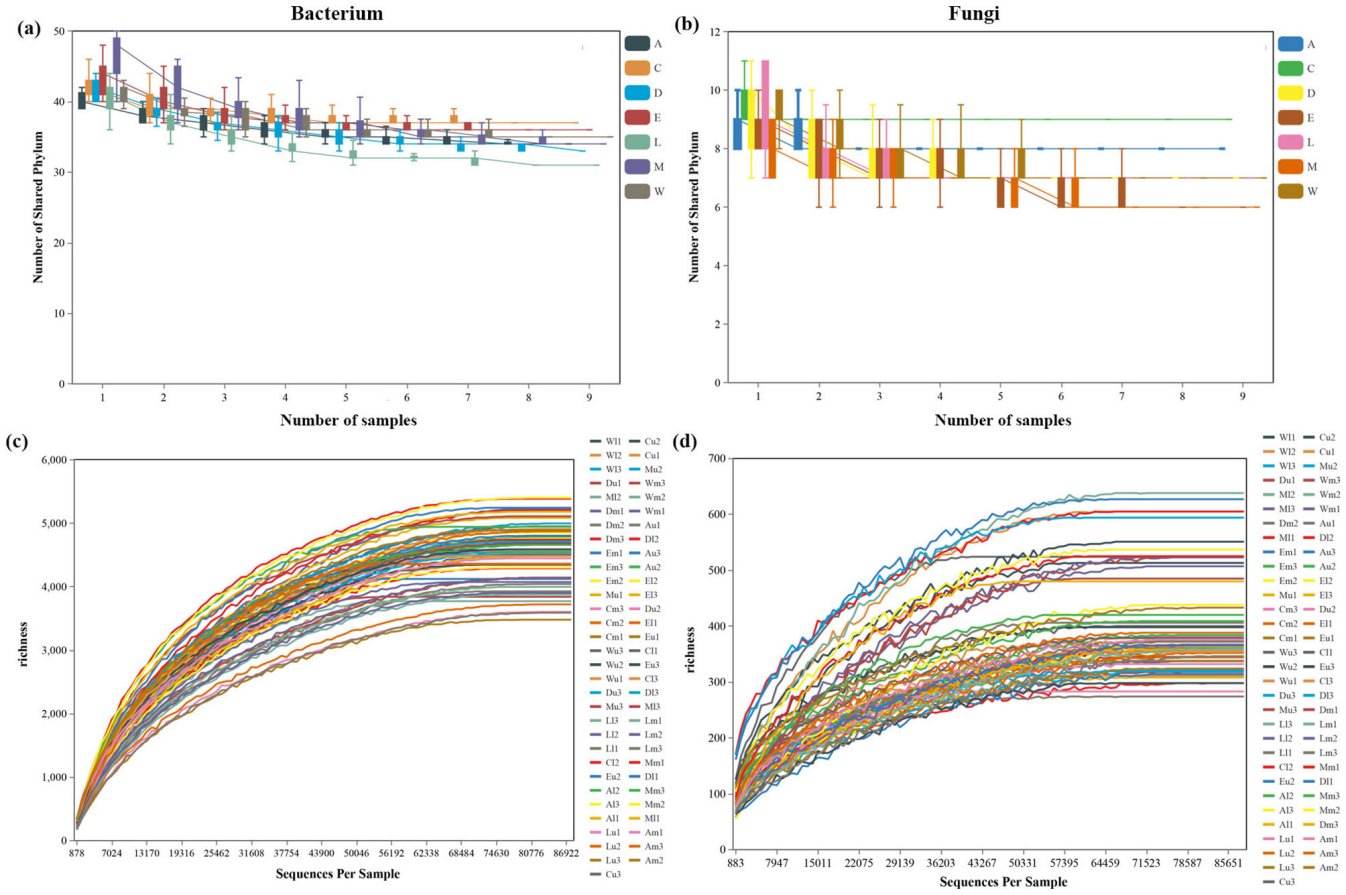
The number of shared phylum across islands as the sample size increases: **(a)** bacteria and **(b)** fungi. Rarefaction curves illustrating the relationship between sequencing depth and species richness across different sample sites for communities: **(c)** bacterial and **(d)** fungal.

We obtained 5,272,525 and 5,273,089 high-quality sequences, yielding 284,934 and 25,321 OTUs from soil bacteria and fungi samples, respectively (Supplementary Tables 2, 3). Bacterial communities exhibited greater variability in the number of shared phyla across the different islands, with this variability increasing as the sample size grew (Figure 2a). In contrast, fungal communities displayed relatively stable and lower diversity, with fewer fluctuations in shared phylum numbers across sites (Figure 2b). The rarefaction curves for both bacterial and fungal communities illustrate the relationship between sequencing depth and species richness across different sample sites. For bacterial communities, species richness increased steadily with increasing sequences per sample, with certain sites reaching a plateau at higher sequencing depths, indicating sufficient sequencing coverage. Sites like Zhaoshu Island (Au, Am, and Al) exhibited more gradual increases in richness, suggesting greater bacterial diversity, while sites such as Cu and Em showed quicker saturation, indicating lower diversity (Figure 2c). Fungal communities followed a similar trend but displayed generally lower richness than bacteria. Sites like Eman coast (Ml, Mm, and Mu) showed gradual increases in fungal diversity, suggesting that more diverse fungal populations required deeper sequencing to be fully represented (Figure 2d). The microbial diversity in areas near *H. arboreum*, such as plots Wuzhizhou Island (W) and Yongxing Island (C), presented a unique pattern. These sites, which experience significant anthropogenic disturbance, showed a decrease in fungal diversity, likely due to the harsh environmental conditions that the plant faces. While bacterial communities maintained relatively high diversity, the reduced fungal diversity reflects the plant's compromised ability to foster a diverse microbial community in such challenging conditions. The quick saturation observed in fungal communities near *H. arboreum* further emphasizes the difficulty the plant faces in maintaining a balanced ecosystem (Figure 2).

**Figure 3:**
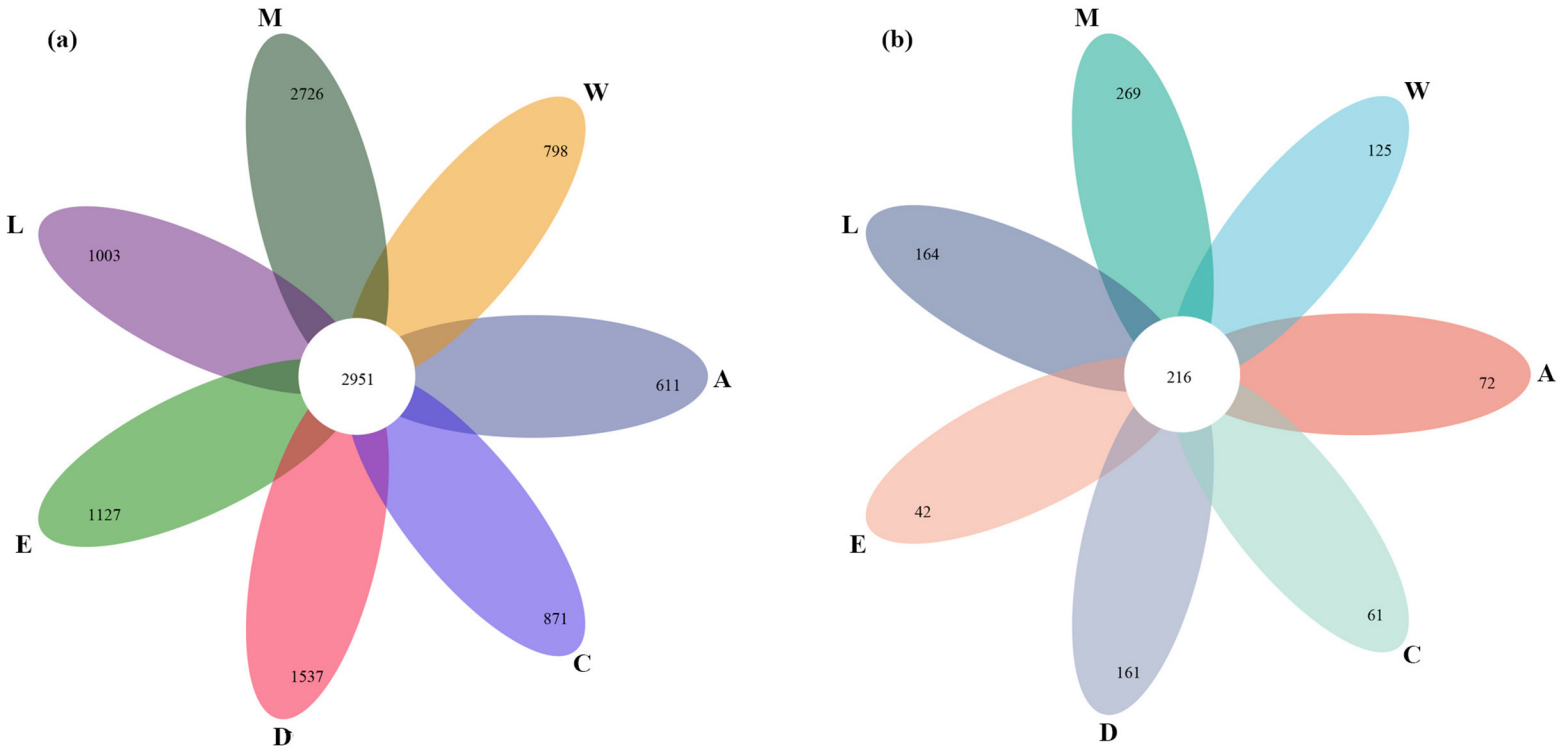
Venn diagrams of diversity across islands: **(a)** bacterial and **(b)** fungal.

Bacterial diversity across the islands is notably high, with a total of 2,951 bacterial species observed. However, some islands, such as Zhaoshu Island (A) and Wuzhizhou Island (W), exhibit relatively lower bacterial diversity, with 611 bacterial species on Zhaoshu Island and 798 species on Wuzhizhou Island (Figure 3a). The diversity of fungi is notably smaller than that of bacteria across the islands, with fewer fungal species observed at each sample plot. Specifically, fungal diversity is lowest on Zhaoshu Island (A), with just 72 fungal species, and Wuzhizhou Island (W) shows 125 fungal species (Figure 3b). The pattern of Eman coast (M) showing the highest fungal diversity aligns with its bacterial diversity, with a total of 269 fungal species observed, while Zhaoshu Island (A) and North Island (E) have notably lower fungal diversity, with 72 fungal species on Zhaoshu Island and 42 species on North Island (E). Overall, bacterial diversity surpasses fungal diversity across the islands, with Eman coast (M) exhibiting the richest diversity in both bacterial and fungal species. On the other hand, islands such as Zhaoshu Island (A) and North Island (E) display low species richness for both bacteria and fungi, particularly in fungi, where North Island (E) has the lowest fungal diversity with just 42 species. Interestingly, the sites near *H. arboreum*, especially in plots on Wuzhizhou Island (W) and Yongxing Island (C), exhibit a distinct microbial diversity pattern. While bacterial diversity remained relatively high, with 798 species on Wuzhizhou Island and 871 species on Yongxing Island (C), fungal diversity was notably lower, with 125 fungal species on Wuzhizhou Island and just 61 fungal species on Yongxing Island. This likely reflects the plant's challenging environmental conditions, which are associated with diminished fungal diversity, particularly in these sites where *H. arboreum* thrives despite harsh conditions. The reduced fungal diversity suggests that the plant's environment is negatively impacting the microbial communities it supports. This supports the notion that *H. arboreum* is facing significant challenges in these environments, as indicated by the compromised fungal diversity, which points to difficulties in sustaining a diverse microbial ecosystem (Figure 3).

### 3.2 Relative abundance and function of microbial groups

**Figure 4:**
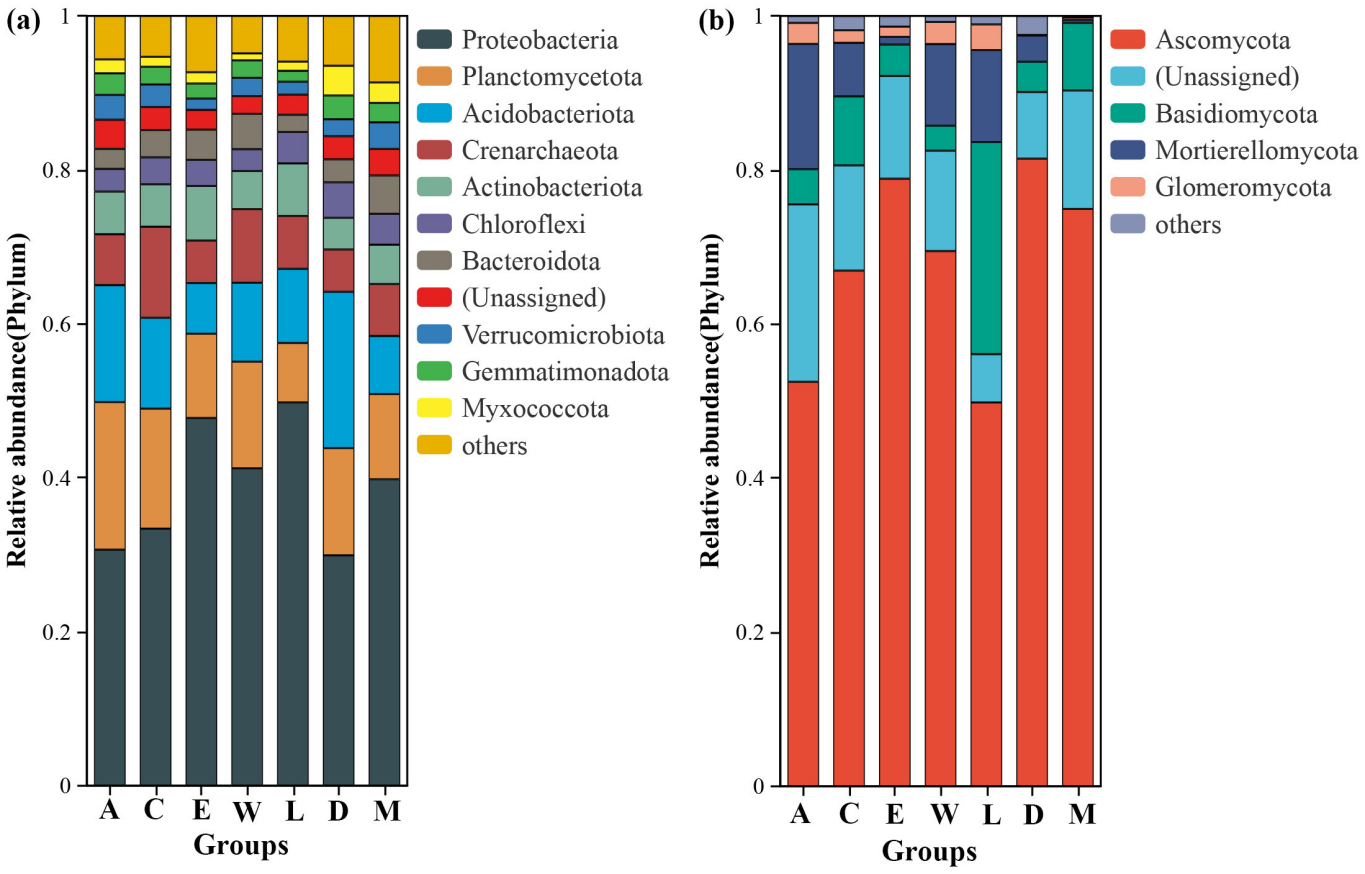
Relative abundance of phyla across different island groups **(a, c, e, w, l, d, m)**: **(a)** bacterial and **(b)** fungal.

Proteobacteria dominate the bacterial communities across the islands, with significant presence of other groups such as Planctomycetota and Acidobacteriota. Across the groups, the relative abundance of Proteobacteria ranges from 0.29 in Plot M (located along the tourist highway in Eman Coast, Danzhou City) to 0.50 in Plot C (Yongxing Island, located in the Xisha Islands). The relative abundance of Planctomycetota varies from 0.08 in Plot D (Danzhou City) to 0.19 in Plot A (Zhaoshu Island, South China Sea). Acidobacteriota ranges from 0.06 in Plot C to 0.20 in Plot E (North Island, Xisha Islands) (Figure 4a). Although the relative abundance of these groups shows slight variation across the islands, Proteobacteria consistently remain the most dominant phylum, particularly in the isolated islands of the Xisha Islands, which have harsh environmental conditions characterized by coral sand. Ascomycota overwhelmingly dominates the fungal communities across the islands, with Ascomycota showing relative abundances ranging from 0.45 in Plot D to 0.79 in Plot E (Figure 4b). Basidiomycota is also present in noticeable proportions, ranging from 0.04 in Plot E to 0.28 in Plot A, and Mortierellomycota shows a range of 0.00 to 0.12 across the different groups. The fungal communities exhibit more variation compared to the bacterial communities, but Ascomycota remains the primary group in all samples. Interestingly, the patterns of microbial diversity near *H. arboreum* (especially in Plot W and Plot C) illustrate a distinct shift, particularly in fungal communities. Plot W is located on Wuzhizhou Island, an independent island near Hainan Island, which has notable tourism development, including coral sand and reefs as dominant features. Plot C, on Yongxing Island, also faces significant human activity. While Proteobacteria remained dominant in these regions, similar to other islands, fungal communities displayed more pronounced changes, especially in the relative abundance of Basidiomycota and Mortierellomycota. In Plot C and Plot W, fungal diversity is reduced, likely due to the harsh environmental conditions and human disturbances surrounding *H. arboreum*. The relatively tall growth of *H. arboreum* in these locations, despite the absence of seedlings, could indicate stress on the microbial ecosystems, especially fungal communities that are more sensitive to environmental changes. The reduced fungal diversity in these areas suggests that *H. arboreum*'s presence in these regions might be associated with compromised microbial ecosystems, particularly concerning fungi, which are more sensitive to environmental stressors (Figure 4, Supplementary Table 4).

**Figure 5:**
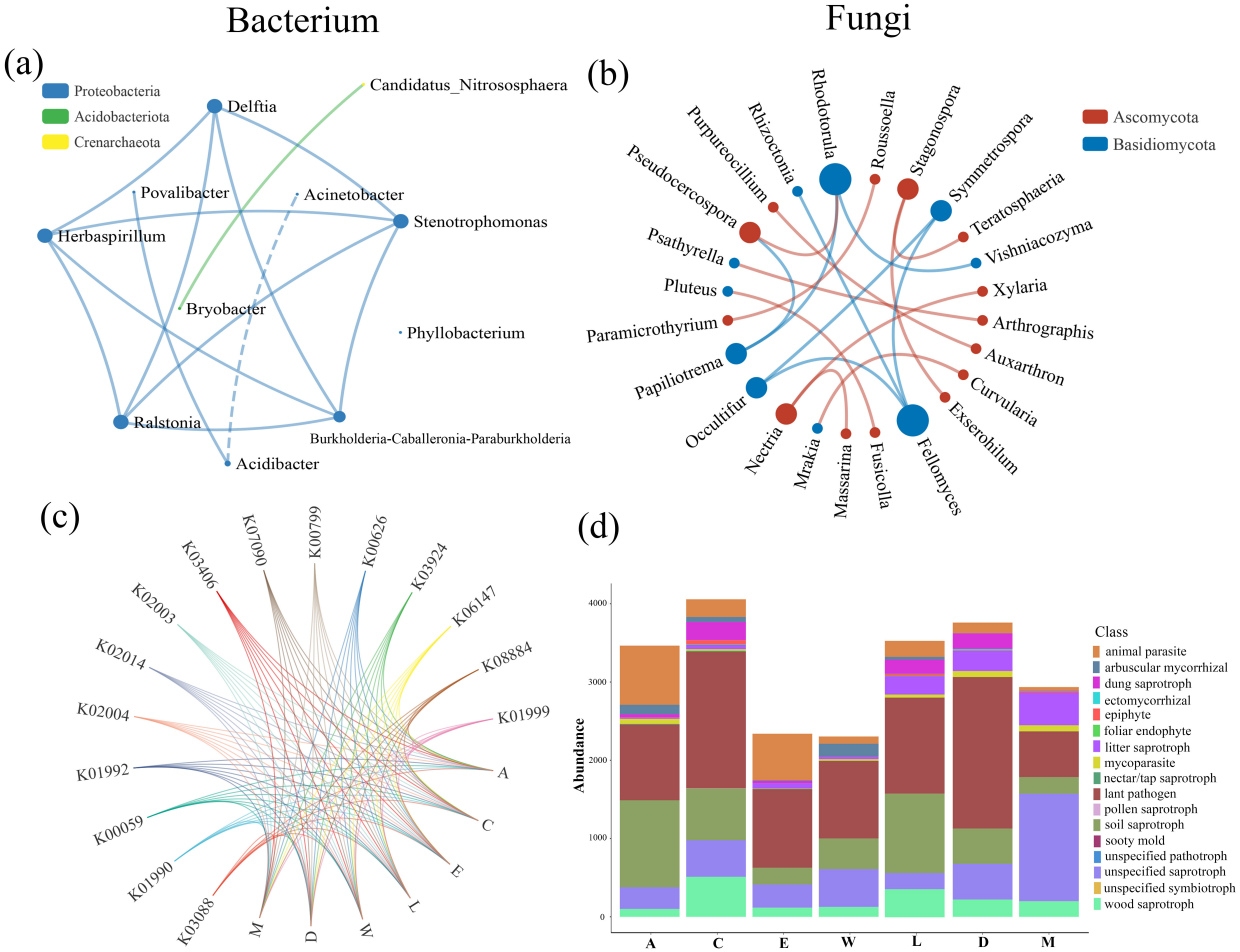
Network analysis and abundance distribution of microbial communities across different island groups: **(a)** bacterial network of genera, **(b)** fungal network of genera, **(c)** bacterial functional gene distribution, **(d)** fungal functional gene distribution.

The network analysis of bacterial taxa, highlighting strong negative correlations between several taxa, such as *Acidibacter* and *Acinetobacter* (*r* = −0.808). These negative relationships suggest competitive or antagonistic interactions, which may have cascading effects on fungal community dynamics, influencing overall ecosystem stability. Such bacterial interactions likely alter the fungal environment, further impacting plant health and regeneration (Figure 5a). Turning to the fungal network analysis in Figure 5b, significant positive correlations are observed between species such as *Stagonospora* and *Exserohilum* (*r* = 0.963), reflecting beneficial symbiotic interactions that contribute to the overall health and stability of the fungal community. These positive relationships are vital for the ecosystem, as they promote nutrient cycling and help maintain the delicate balance of plant-fungal interactions necessary for plant growth and regeneration (Figure 5b). Regarding functional diversity, Figure 5c illustrates the variation in bacterial communities across different plots. For instance, Plot A demonstrates high levels of Proteobacteria, particularly Povalibacter and Herbaspirillum, with relative abundances of 0.003393 and 0.005364, respectively. These bacteria play crucial roles in nitrogen fixation and soil health, promoting plant growth and enhancing soil structure, which benefits overall ecosystem functioning. Povalibacter, for instance, is known for its ability to promote nutrient cycling, while Herbaspirillum contributes to plant growth promotion. In contrast, Plot W highlights the negative impact of human disturbance on bacterial community structure. Elevated levels of Ralstonia (0.003287) and Acinetobacter (0.004158) are observed. These bacteria are often associated with stressed environments due to anthropogenic disturbances, such as pollution and nutrient imbalances. The presence of such bacteria in Plot W suggests that human activity is impairing the bacterial community, leading to challenges in soil health and plant regeneration. Despite the survival of mature plants, the altered bacterial community structure in Plot W is associated with inhibited regeneration of *H. arboreum*. On the other hand, Figure 5d illustrates the variation in fungal communities across the same plots. In Plot A, we observe high levels of arbuscular mycorrhizal fungi (1,210.8) and dung saprotrophs (405.9), both of which are essential for nutrient cycling, organic matter decomposition, and plant health. Arbuscular mycorrhizal fungi, for instance, have a key role in enhancing nutrient uptake by plant roots, while dung saprotrophs help break down organic matter, enriching the soil and supporting plant growth. The abundance of these fungi in Plot A supports a thriving ecosystem with efficient nutrient cycling. In stark contrast, Plot W shows elevated levels of animal parasites (922) and plant pathogens (1,349.4), suggesting that human activities, such as tourism development, have disrupted the fungal community structure. The presence of these harmful fungi indicates a negative impact on plant health and regeneration. The increased abundance of animal parasites and plant pathogens in Plot W reflects the degradation of ecosystem health and the inability of *H. arboreum* to regenerate, despite the persistence of mature plants. Together, these results emphasize the critical importance of minimizing human disturbance to preserve fungal communities. Fungi, as essential players in nutrient cycling and plant health, are vital for the overall stability and regeneration of ecosystems, and their protection is crucial for maintaining biodiversity (Figure 5).

### 3.3 Alpha diversity and beta diversity

**Figure 6:**
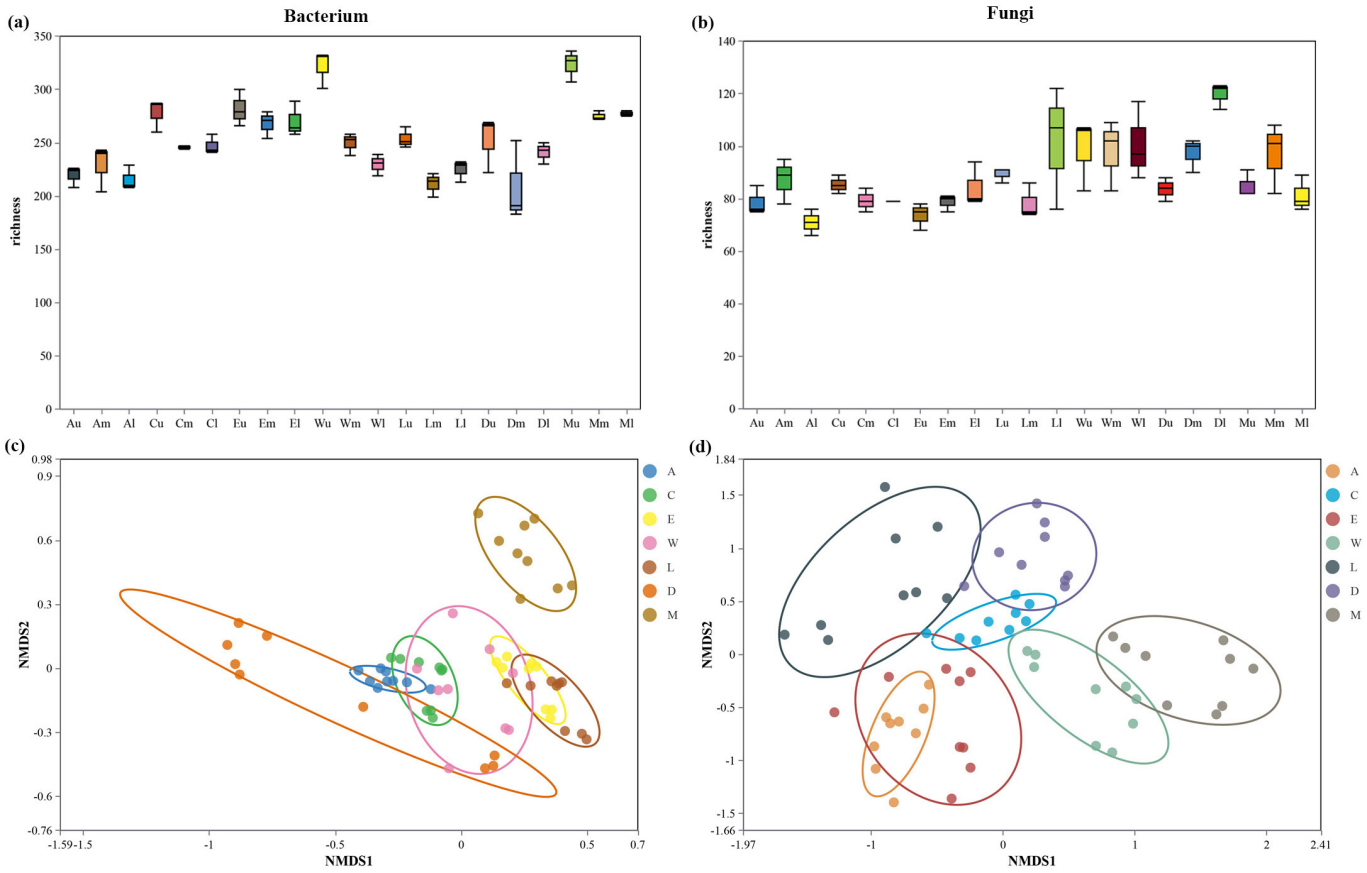
**(a, b)** Alpha diversity Box diagram of difference analysis between groups. **(c, d)** beta diversity. Non-metric multidimensional scaling (NMDS) analysis of microbial communities across different island groups.

The bacterium richness was observed to be highest in the topsoil of Zhaoshu Island (Plot A, topsoil layer) with a richness value of 350, compared to the lowest bacterium richness in Eman Coast low soil (Plot D, subsoil) at 190. Intermediate values were found in the coastal plots, with Wuzhizhou Island medium soil (Plot W) showing 240 and Lingao County middle soil (Plot L) showing 210. This pattern indicates that more pristine environments, such as Zhaoshu Island, support a greater diversity of bacterium species, while disturbed sites, such as those influenced by human activity (e.g., Wuzhizhou Island and Eman Coast), exhibit lower bacterium richness (Figure 6a). Fungi richness showed a similar distribution across the plots. The highest fungi richness was recorded in Plot Am (middle soil of Zhaoshu Island) at 130, while the lowest fungi richness was observed in Eman Coast low soil (Plot D, subsoil), which had a value of 70. Wuzhizhou Island medium soil (Plot W) exhibited a fungi richness of 90, demonstrating a moderate richness despite the human influence in this area. This suggests that fungi diversity is also sensitive to the balance between environmental conditions and human impacts (Figure 6b). Non-metric multidimensional scaling (NMDS) analysis of bacterium communities revealed distinct clustering patterns. Plots with minimal human interference, such as Zhaoshu Island, Yongxing Island, and North Island, exhibited tight clusters, with a stress value of 0.1, indicating relatively stable microbial community structures. In contrast, plots affected by human activity, such as Wuzhizhou Island (Plot W) and Eman Coast, showed more dispersed clusters, with higher stress values of around 0.2. This dispersion suggests that human disturbances, particularly from tourism, have altered the microbial communities, leading to more diverse but less cohesive bacterium compositions (Figure 6c). Similarly, NMDS analysis of fungi communities indicated clear separation between pristine and disturbed environments. Zhaoshu Island (Plot A) and Yongxing Island (Plot C) clustered together with low stress values of 0.12, reflecting stable fungi communities. However, Wuzhizhou Island (Plot W), despite its high fungi richness, showed a higher stress value of 0.25, which highlights the significant impact of human development on the fungi community structure. The more disturbed plots, like Eman Coast, had increased dispersion and stress, reinforcing the idea that human activities, such as tourism, can disrupt the natural balance of microbial communities (Figure 6d). The analysis of *H. arboreum* distribution reveals a noteworthy trend. While this species grows well in disturbed environments, particularly in Plot W (Wuzhizhou Island) and Plot C (Yongxing Island), no seedlings have been observed in these areas. Despite the towering stature of the mature plants, the absence of seedlings suggests a disruption in the plant's natural regeneration process. This is consistent with the microbial community findings, where human activity appears to foster conditions for mature plants but impedes the ecological processes necessary for seedling establishment (Figure 6).

### 3.4 Multi-group comparative analysis of species differences

**Figure 7:**
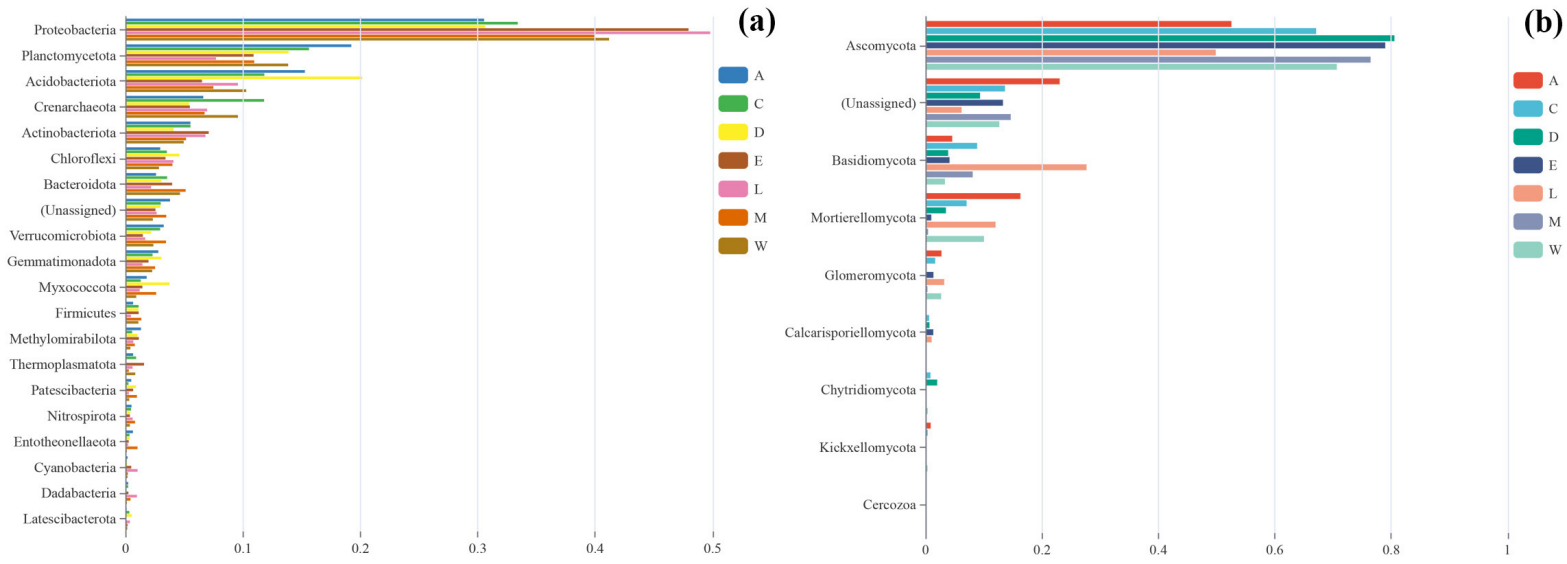
Bar charts comparing the relative abundance of phyla across various sample sites: **(a)** bacterial and **(b)** fungal.

The microbial community composition across the different plots reveals distinct variations that are influenced by both environmental conditions and human activity. Proteobacteria is the predominant bacterial taxon across all sites, with the highest relative abundance in Plot L at 0.498, followed by Plot E at 0.480. The abundance of Proteobacteria decreases slightly in more disturbed plots like Plot A (0.305) and Plot D (0.306), but it remains a dominant feature across all sites (Figure 7a). Other bacterial taxa, such as Planctomycetota and Acidobacteriota, show variable presence across the sites, with Acidobacteriota reaching a high of 0.201 in Plot D (Danzhou City), but being less abundant in Plot L (Lingao Coast) (0.096). The fungal community is similarly dominated by Ascomycota, which is most abundant in Plot D (0.805), followed by Plot E (0.790) and Plot M (0.764). Basidiomycota and Mortierellomycota are present in lower proportions, with Basidiomycota reaching its highest in Plot C (Yongxing Island) (0.088) and Plot M (Eman Coast) (0.081), while Mortierellomycota is more abundant in Plot A (0.163) and Plot L (0.120) (Figure 7b). Despite the abundant growth of *H. arboreum* in these areas, especially in Plot W and Plot C, there is a notable absence of seedling specimens, suggesting that human development and disturbance may be hindering the reproductive success of this species. This contrasts with the more natural and less disturbed environments in Plot A and Plot C, where the microbial communities remain relatively stable, but still, *H. arboreum* seedlings are absent. These findings underscore the complex relationship between environmental factors, human activity, and microbial communities, with human disturbance playing a significant role in shaping the microbial diversity and potentially affecting the ecological dynamics of *H. arboreum* populations (Figure 7).

### 3.5 Environmental influence on microbial communities surrounding *Heliotropium arboreum*

**Figure 8:**
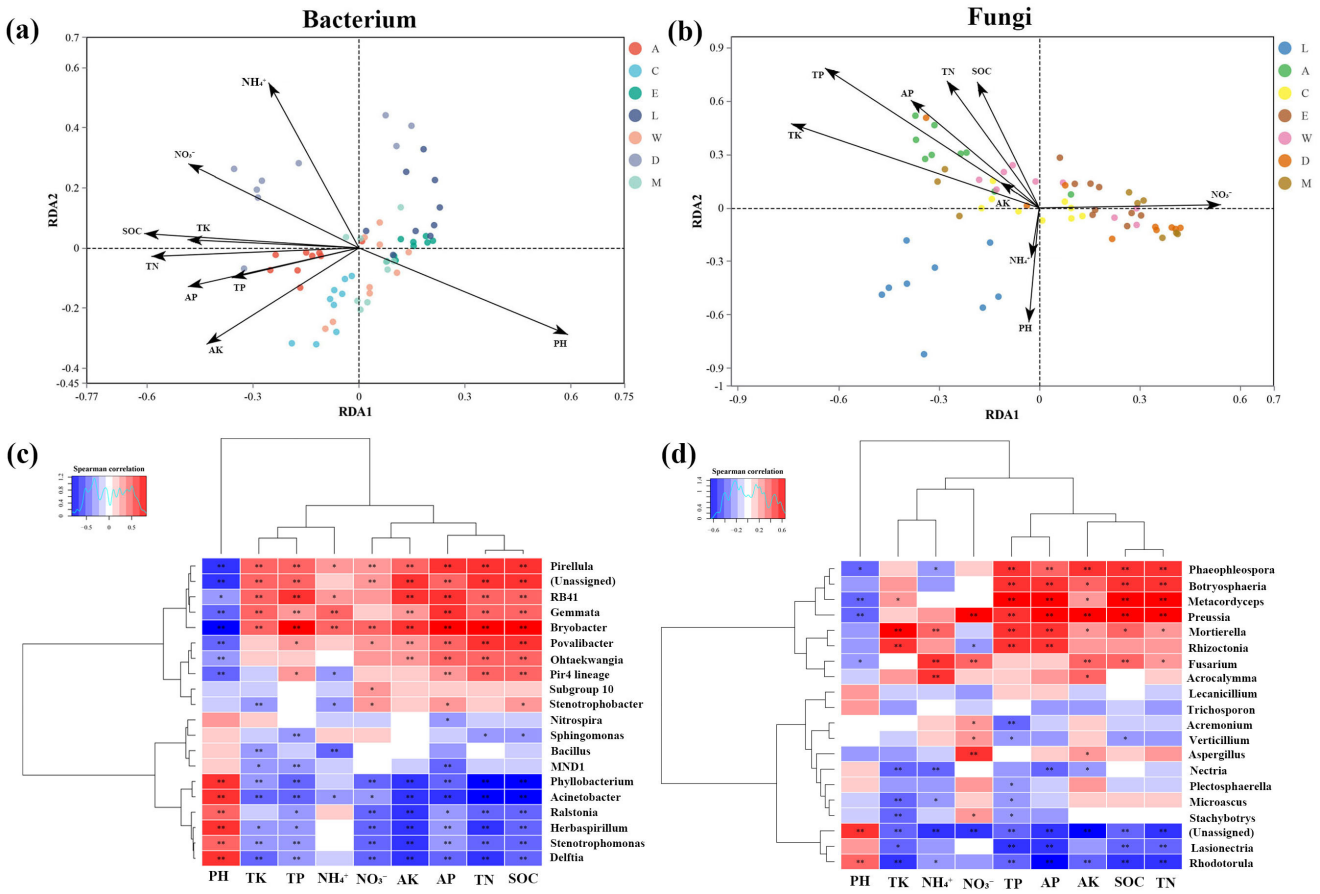
Redundancy Analysis (RDA) and correlation heatmaps of microbial communities across islands: **(a)** RDA plot for bacterial communities with environmental factors. **(b)** RDA plot for fungal communities with environmental factors. **(c)** Correlation heatmap for bacterial communities and their relationship to environmental factors. **(d)** Correlation heatmap for fungal communities and their relationship to environmental factors.

The RDA and heatmap analyses of bacterial and fungal communities reveal significant environmental influences shaping microbial populations in the areas surrounding *H. arboreum*. The bacterial RDA (Figure 8a) highlights the strong impact of factors such as pH, ${\text{NH}}_{4}^{+}$, and TP on bacterial community composition, with distinct clustering observed between plots, particularly Zhaoshu Island (A) and Yongxing Island (C), which share similar microbial profiles, while other plots like Eman coast (M) and Lingao coast (L) are more differentiated. Similarly, the fungal RDA (Figure 8b) shows how environmental variables like ${\text{NO}}_{3}^{-}$ and AK affect fungal community structure, with notable separation between plots influenced by human activity and those in more isolated environments. While the heatmap shows two asterisks, the results in the tables include three levels of significance. The bacterial heatmap highlights strong positive correlations between specific bacterial genera and higher nutrient availability in the microbial communities around *H. arboreum*. Notably, *Bryobacter* (0.810^***^) and *Stenotrophobacter* (0.496^***^) exhibit significant correlations, indicating a marked abundance of these genera in nutrient-rich environments. Similarly, *Pirellula* (0.639^***^) and *Phyllobacterium* (0.708^***^) show high positive correlations with nutrient availability, further suggesting that these genera thrive under enriched conditions. Conversely, some genera, such as *Stenotrophomonas* (-0.488^***^), display negative correlations, highlighting their reduced abundance in nutrient-dense environments (Figure 8c, Supplementary Table 5). The fungal community heatmap reveals a similar pattern in the microbial communities surrounding *H. arboreum*, with specific genera correlating strongly with nutrient levels, including nitrate (${\text{NO}}_{3}^{-}$) and total phosphorus (TP). *Preussia* (0.585^***^) and *Metacordyceps* (0.616^***^) are particularly abundant in nutrient-rich areas, indicating their positive association with higher nutrient availability. This suggests their potential role in organic matter degradation or symbiotic relationships with plant roots to enhance nutrient absorption. Other fungal genera, such as *Mortierella* (0.331^**^) and *Fusarium* (0.285^*^), also show significant positive correlations with nutrient levels, reinforcing the link between nutrient richness and fungal community structure (Figure 8d, Supplementary Table 5).

## 4 Discussion

The microbial communities associated with *H. arboreum* exhibit clear differentiation between bacterial and fungal populations in response to environmental factors. Bacterial communities showed greater diversity than fungal ones, likely due to their higher adaptability to environmental stressors such as nutrient availability and pH fluctuations. This aligns with previous studies indicating that bacteria are generally more resilient to environmental changes than fungi (Lauber et al., 2008; Loos et al., 2024). Bacterial communities vary significantly across environments, influenced by a combination of factors including soil type, nutrient availability, and environmental stress. Among these, soil pH is particularly influential: acidic soils tend to support fewer bacterial species than neutral or alkaline soils. For example, farmland soils with higher pH levels typically harbor a more diverse bacterial community than acidic forest soils, a trend supported by studies linking pH to bacterial composition (Kim et al., 2021). Nutrient availability also shapes community structure; nutrient-rich environments favor copiotrophic bacteria, while nutrient-poor conditions select for oligotrophic, stress-tolerant species. Additional environmental pressures such as temperature fluctuations and varying water availability further influence bacterial adaptations, especially on isolated islands where such factors are often more pronounced. For instance, alpine soils subjected to nitrogen enrichment show shifts in bacterial community structure, with pH playing a central mediating role (Yuan et al., 2016). Anthropogenic influences, including nutrient enrichment and pollution, can further alter these communities. In aquaculture zones, for example, nutrient loading increased bacterial diversity but also changed community composition due to stressors such as hypoxia and ocean acidification (Ng and Chiu, 2020). Overall, bacterial community composition and resilience are closely tied to the interplay of environmental variables, which can differ significantly across locations particularly in island ecosystems. In contrast, fungal diversity was lower in areas near *H. arboreum*, especially in more disturbed plots. This may reflect the plant's sensitivity to human activity and extreme climatic conditions, which also adversely affect associated fungal communities. Fungi are often more specialized and less adaptable than bacteria, making them more susceptible to environmental disturbances (Kaisermann et al., 2015). On Wuzhizhou Island, where fungal diversity near *H. arboreum* is particularly low, environmental stressors likely have a disproportionate impact on fungi.

Mycorrhizal and saprotrophic fungi, in particular, depend on specific nutrient conditions and are sensitive to environmental shifts. Factors such as nutrient depletion, temperature extremes, and pollution can significantly disrupt fungal populations. For example, exposure to pollutants like heavy metals and hydrocarbons can impair fungal metabolism, especially in saprotrophic species critical to nutrient cycling (Mikryukov and Dulya, 2018). Moreover, fungi are more sensitive than bacteria to pH fluctuations and soil moisture variability. Extreme changes in these factors can severely impact fungal communities, while bacteria, with their higher adaptability and faster growth rates, often tolerate such stressors more effectively (Xu et al., 2023). Fungi also require specific environmental cues for spore germination and growth, further limiting their ability to adapt to rapidly changing conditions. In contrast, bacteria can proliferate quickly under a wide range of circumstances (Tortella et al., 2005). The reduced fungal diversity observed near *H. arboretum* particularly in disturbed environments like Wuzhizhou Island likely results from the specialized nature of fungi and their heightened sensitivity to stressors such as nutrient loss, temperature extremes, pollution, and soil condition variability.

Microbial communities associated with *H. arboreum* in Hainan's coastal ecosystems are shaped by a combination of environmental stressors and anthropogenic influences. Bacterial populations are primarily dominated by members of the phylum Proteobacteria, known for their metabolic versatility in nutrient-poor, saline soils where key processes such as nitrogen fixation and organic matter decomposition are critical (Fierer et al., 2007; Leal et al., 2024). Other important bacterial contributors include Planctomycetota, which excel in phosphorus scavenging within oligotrophic coral sands, and Acidobacteriota, which thrive in acidic, low-carbon environments (Bergkemper et al., 2016). Interactions among bacterial taxa particularly competitive and antagonistic dynamics play a pivotal role in microbial ecosystem stability and in shaping fungal community structure. For example, competition between genera such as *Acidibacter* and *Acinetobacter* can lead to shifts in resource availability, directly impacting the establishment and composition of fungal communities (Wang and Kuzyakov, 2024). Highly competitive bacteria like *Acinetobacter* may outcompete fungi for key nutrients such as nitrogen and organic matter, resulting in imbalances within the microbial community. These bacterial–fungal interactions can influence fungal communities through both inhibitory and facilitative mechanisms. On one hand, bacteria can suppress fungal growth by outcompeting them for limited resources or producing antifungal compounds. On the other hand, synergistic interactions between certain bacterial and fungal taxa may promote fungal establishment and enhance nutrient cycling beneficial outcomes for plant health and regeneration under stress conditions (Bennett and Feibelman, 2001). For instance, bacteria can indirectly support fungi by inhibiting other microbial competitors, or by producing metabolites such as surfactin, which alters fungal cell walls and shifts ecological niches (Richter et al., 2024). Fungi, in turn, can modulate bacterial populations through the production of antimicrobial compounds, establishing a dynamic interplay of competition and cooperation. Under environmental stress such as nutrient depletion, temperature extremes, or pollution these interactions become more pronounced. Both bacteria and fungi may shift their strategies for resource acquisition in response, with outcomes that can either support or hinder plant regeneration. For example, in contaminated soils, bacteria may dominate, disrupting beneficial fungal-bacterial synergies essential for nutrient cycling (Shen et al., 2023). Conversely, under certain conditions, bacterial facilitation of fungal growth may enhance plant regeneration by improving nutrient availability or modifying soil conditions in favor of plant survival.

Fungal communities associated with *H. arboreum* are primarily dominated by stress-tolerant Ascomycota, which are well-adapted to harsh coastal environments due to their melanized hyphae that provide resistance against desiccation and UV radiation. Basidiomycota contribute sporadically, primarily in localized, organic-rich microhabitats where they facilitate lignin decomposition (Tedersoo et al., 2014). However, environmental stressors particularly nitrogen limitation and pH fluctuations have a disproportionately negative impact on fungal diversity, often leading to a competitive advantage for more resilient bacterial taxa (Rousk et al., 2010). These stressors significantly affect key fungal functional groups, notably mycorrhizal fungi and pathogenic fungi. Nitrogen limitation, for instance, can suppress the colonization of plant roots by ectomycorrhizal fungi, which play a crucial role in nutrient cycling. Species within the genus *Cortinarius*, for example, specialize in the uptake of organic nitrogen from soil. When nitrogen levels increase, fungal community composition shifts favoring species that utilize inorganic nitrogen over those adapted to organic nitrogen sources (Hobbie et al., 2009). This transition often results in decreased efficiency in nutrient cycling, as specialized decomposers are replaced by less effective generalists. Soil pH fluctuations also play a major role in shaping fungal communities. Fungal diversity tends to peak in neutral to slightly acidic soils, a trend particularly evident in mycorrhizal fungi. For instance, studies in oak savannas have shown that nitrogen enrichment can alter soil pH and subsequently shift fungal community composition, favoring species like *Russula* that are better adapted to more alkaline conditions (Avis and Charvat, 2005). Such shifts can disrupt plant-fungal symbioses, negatively affecting plant nutrient acquisition and overall health. In contrast, pathogenic fungi such as *Fusarium* and *Alternaria* often thrive in disturbed environments, like those found on Wuzhizhou Island. These opportunistic fungi benefit from human-induced stressors including pollution, soil compaction, and nutrient imbalances. Elevated nitrogen levels and organic waste from human activity can promote the proliferation of these pathogens (Jirout et al., 2011). Their dominance in disturbed areas poses a direct threat to *H. arboreum*, as they can impair plant growth, weaken defense mechanisms, and hinder regeneration. The increased prevalence of pathogenic fungi under anthropogenic stress exacerbates plant vulnerability by disrupting nutrient uptake and reducing resilience to further environmental challenges (Di Martino et al., 2022). In contrast to mycorrhizal fungi, which are highly sensitive to shifts in nitrogen availability and pH, pathogenic fungi are more adaptable and opportunistic in degraded habitats. This imbalance contributes to ecosystem dysfunction, particularly in already stressed plant species like *H. arboreum*.

The alpha and beta diversity patterns of microbial communities associated with *H. arboreum* reveal the intricate interplay between environmental gradients and anthropogenic pressures in Hainan's coastal ecosystems. Alpha diversity analyses indicated that bacterial richness was highest in the topsoil of Zhaoshu Island (Plot Au), likely due to nutrient enrichment from plant-derived organic debris and root exudates. This topsoil layer supports diverse bacterial communities largely because of favorable soil conditions particularly higher organic matter content, which serves as a rich source of carbon and nutrients, promoting microbial proliferation and diversity (Uksa et al., 2017). Additionally, root exudates contribute significantly to shaping bacterial community structure by providing accessible carbon compounds, which favor taxa adapted to metabolize these substrates, enhancing nutrient cycling and soil fertility (Xiao et al., 2024). In contrast, fungal richness peaked in the middle soil layer of Plot Am, likely reflecting vertical niche partitioning within the soil profile. Fungi, being more specialized than bacteria, often depend on complex organic substrates such as decomposed plant matter, which may be more abundant in intermediate soil layers (Preusser et al., 2019). Moreover, the middle layer often maintains more stable moisture and temperature conditions, which are essential for fungal growth. Reduced microbial competition in this layer compared to the more nutrient-rich and dynamic topsoil may also allow fungi to establish more diverse communities, as bacteria tend to dominate upper layers under such conditions (Wang et al., 2024a). This niche partitioning has important ecological implications. Bacteria in the topsoil typically thrive on simpler, more labile carbon sources, while fungi in deeper layers specialize in breaking down more complex, recalcitrant materials. This functional differentiation minimizes competition and supports coexistence, contributing to overall soil ecosystem stability. Together, bacteria and fungi play complementary roles in nutrient cycling: bacteria drive rapid turnover of readily available nutrients, while fungi decompose more complex substrates, releasing essential nutrients that sustain long-term soil fertility and plant growth (Wang et al., 2023; Lladó et al., 2018). By contrast, microbial richness was lowest at the Eman Coast (Plot M), likely due to poor soil quality, nutrient leaching in sandy substrates, and frequent human trampling that disrupts microbial habitats (Delgado-Baquerizo et al., 2020). These disturbances result in less hospitable conditions for both bacterial and fungal communities, leading to reduced diversity and ecosystem function. Beta diversity, assessed using non-metric multidimensional scaling (NMDS), showed clear community clustering: microbial assemblages on relatively pristine islands (Zhaoshu and Yongxing) formed distinct groups, separated from those at human-impacted sites like Wuzhizhou Island and the Eman Coast. Interestingly, disturbed plots exhibited higher microbial richness, but this richness was largely due to the proliferation of ruderal taxa, including opportunistic bacteria and pathogenic fungi. These taxa thrive in homogenized, stressed environments and often contribute less to long-term ecosystem stability. This paradox higher diversity but lower ecological integrity aligns with global findings that anthropogenic disturbances decouple microbial diversity from ecosystem function, as observed in degraded mangroves and overused grasslands (Fierer et al., 2007; Crowther et al., 2015).

Human disturbances, such as tourism and land-use changes, significantly hinder the regeneration of native plant species like *H. arboreum*, particularly in impacted areas such as Wuzhizhou Island and the Eman Coast. These disturbances often result in alterations to soil structure and microbial community composition especially fungal communities that are critical to seedling establishment and ecosystem recovery. Soil compaction, nutrient imbalances, and the introduction of pathogens create hostile conditions for seedlings by disrupting root–microbe interactions and reducing soil fertility (Domínguez-Begines et al., 2020). The absence of *H. arboreum* seedlings in disturbed sites suggests that these changes directly limit the species' ability to regenerate. Fungal communities are especially sensitive to such environmental disruptions. As key players in nutrient cycling and symbiotic root associations, fungi are affected by shifts in soil pH, organic matter content, and moisture levels (Bertacchi et al., 2016). In disturbed soils, fungal communities often shift toward pathogenic taxa, increasing disease pressure on seedlings. For example, soil-borne pathogens like *Phytophthora cinnamomi* have been shown to dramatically reduce seedling survival in degraded habitats (Domínguez-Begines et al., 2020). This imbalance marked by a decline in mutualistic fungi and a rise in pathogenic species compromises the soil's capacity to support native plant regeneration. Moreover, the proliferation of pathogens such as *Fusarium* and *Alternaria* in disturbed environments correlates with increased seedling mortality (Kennedy and Egger, 2010). These conditions are often compounded by reduced organic matter and increased soil erosion, which further limit nutrient availability and establish a negative feedback loop that restricts plant recovery. Tourism-related activities exacerbate these effects by compacting the soil, reducing its porosity and moisture retention both essential for microbial viability and seedling development (Zhang et al., 2011). Additionally, trampling, habitat fragmentation, and the introduction of exotic species further degrade native microbial networks and plant recruitment capacity.

Multi-group comparative analyses revealed significant differences in microbial community structures across *H. arboreum* habitats, shaped by localized environmental conditions. Bacterial communities were consistently dominated by Proteobacteria, a phylum well-adapted to nutrient-poor, coastal soils where nitrogen cycling and organic matter decomposition are key ecological processes (Fierer et al., 2007; Wang et al., 2024b). Other taxa, including Planctomycetota, Acidobacteriota, and Crenarchaeota, exhibited site-specific distributions: Planctomycetota thrived in phosphorus-limited coral sands on Zhaoshu Island, facilitating the breakdown of recalcitrant organic compounds (Bergkemper et al., 2016), while Acidobacteriota were abundant in acidic, low-carbon soils of isolated islands (Jones et al., 2009). Crenarchaeota, adapted to oligotrophic conditions, were enriched in deeper soils of pristine sites, reflecting their ecological specialization for nutrient-scarce environments (Spang and Ettema, 2017). Fungal communities were predominantly composed of Ascomycota, which are stress-tolerant and capable of decomposing resistant plant litter through melanized hyphae (Tedersoo et al., 2014). Basidiomycota and Mortierellomycota displayed niche preferences, being more abundant in moist, organic-rich soils at Eman and Lingao coasts, where they likely contribute to lignin degradation and organic phosphorus mobilization (Floudas et al., 2012; Nguyen et al., 2016). However, in tourist-impacted sites, the reduced abundance of Acidobacteriota and Mortierellomycota reflects a broader trend in which nutrient enrichment destabilizes specialist microbial taxa (Delgado-Baquerizo et al., 2020). These shifts emphasize how soil type, nutrient gradients, and human activity collectively influence microbial composition and, by extension, the resilience of *H. arboreum*.

Environmental drivers shaping these microbial communities were further clarified through redundancy analysis (RDA) and heatmap visualizations. These analyses highlighted the roles of soil pH, ammonium (${\text{NH}}_{4}^{+}$), nitrate (${\text{NO}}_{3}^{-}$), and total phosphorus (TP) in structuring both bacterial and fungal assemblages. RDA showed that bacterial communities in pristine sites like Zhaoshu (A) and Yongxing (C) clustered together due to shared characteristics—neutral pH and oligotrophic, coral-derived sands with low nitrogen content. These environments supported stress-adapted taxa such as Acidobacteriota and Planctomycetota (Fierer and Jackson, 2006; Delgado-Baquerizo et al., 2017). In contrast, Eman Coast (M) and Lingao Coast (L), which receive higher nutrient inputs from human activity, favored copiotrophic genera such as *Byobacter* and *Stenotrophobacter*, which thrive under enriched conditions (Daleo et al., 2018). Fungal community structure followed similar patterns. Ascomycota (e.g., *Preussia*) were dominant in nitrogen-poor sites, whereas nutrient-rich areas supported genera such as *Metacordyceps*, known for their roles in decomposition and potential pathogenicity (Urbanová et al., 2015). Heatmap analyses also demonstrated the sensitivity of fungal taxa to pH and nitrogen availability: for instance, Basidiomycota declined in acidic, high-${\text{NH}}_{4}^{+}$ environments, consistent with their preference for stable, organic-rich substrates (Tedersoo et al., 2014). Human disturbances intensified these shifts, leading to homogenized microbial communities with reduced functional diversity. This trend mirrors global patterns in which nutrient enrichment and habitat degradation disrupt microbial symbioses critical for plant resilience (Delgado-Baquerizo et al., 2020). The evidence underscores the urgent need for targeted conservation strategies aimed at reducing nutrient loading and rehabilitating degraded habitats. Protecting microbial diversity and function is essential for sustaining the regeneration and long-term survival of *H. arboreum* in coastal ecosystems.

## 5 Conclusions

This study provides a thorough examination of the microbial community composition and diversity surrounding *H. arboreum* across several islands in the South China Sea and Hainan Province. The results underscore the substantial influence of environmental factors, including soil pH, nitrogen availability, and phosphorus levels, on the microbial landscape in these regions. Bacterial communities, dominated by Proteobacteria, exhibited higher diversity compared to fungal communities, which were primarily composed of Ascomycota. The findings revealed clear separations in bacterial communities between islands, with greater clustering observed for fungal populations, suggesting the role of environmental variability and geographical isolation in shaping microbial diversity. Redundancy analysis (RDA) further confirmed that nitrogen levels and soil pH are key drivers in structuring microbial communities, emphasizing the importance of nutrient availability in fostering microbial diversity. The use of heatmap and rarefaction curve analyses highlighted how environmental stressors influence microbial richness, demonstrating the need for sufficient sequencing depth to fully capture microbial diversity. These insights are crucial for understanding the ecological dynamics of *H. arboreum*, especially as the plant thrives in areas with higher microbial diversity, such as Eman coast (M), and faces challenges in regions with lower diversity, like Zhaoshu Island (A). The study highlights the critical role of environmental factors in shaping the microbial communities associated with *H. arboreum*, which is vital for developing effective conservation and ecological management strategies in these fragile island ecosystems.

## Data Availability

The original contributions presented in the study are publicly available. This data can be found here: https://doi.org/10.6084/m9.figshare.29402513.v1.
