# Supplementary material for: Ecological dynamics and microbial community composition of Heliotropium arboreum in the coastal ecosystems of Hainan province
Source: Front Microbiol. 2025 Jul 3;16:1611262. doi: 10.3389/fmicb.2025.1611262 (PMC12267182; doi:10.3389/fmicb.2025.1611262)
Supplement: Supplementary file 1 [file Table_1.docx]

Supplementary Material

# Supplementary and Tables

Table s1 Determination of soil physical and chemical properties

| **Samples** | **TN** | **TP** | **TK** | **AP** | **AK** | **NH4+** | **NO3** | **SOC** | **PH** |
| --- | --- | --- | --- | --- | --- | --- | --- | --- | --- |
| Au1 | 6.2592 | 897.3930 | 3.7562 | 12.2461 | 95.94 | 3.9561 | 0.9997 | 80.7807 | 8.158 |
| Au2 | 5.8958 | 771.1023 | 2.9841 | 13.7424 | 90.39 | 3.9270 | 0.9040 | 75.8893 | 8.188 |
| Au3 | 5.9003 | 863.9602 | 5.7413 | 14.3540 | 100.2 | 4.5384 | 0.8642 | 78.2734 | 8.109 |
| Am1 | 7.7158 | 782.5872 | 3.8086 | 11.1846 | 83.68 | 5.2649 | 1.8208 | 106.6442 | 8.166 |
| Am2 | 7.2688 | 872.2867 | 3.1101 | 11.9782 | 90.56 | 4.3924 | 2.9209 | 103.3527 | 8.14 |
| Am3 | 8.1965 | 754.7153 | 2.9171 | 10.2494 | 88.18 | 3.1413 | 2.6658 | 111.0345 | 8.17 |
| Al1 | 6.2902 | 648.3566 | 2.5874 | 8.5307 | 56.62 | 4.0871 | 9.5057 | 99.3476 | 8.283 |
| Al2 | 7.7841 | 706.8806 | 3.8657 | 8.1364 | 50.81 | 3.8543 | 10.3508 | 102.0193 | 8.352 |
| Al3 | 6.0147 | 690.7845 | 2.9593 | 8.1213 | 51.82 | 5.0619 | 11.7459 | 102.1292 | 8.281 |
| Cu1 | 4.4279 | 5.4034 | 0.7374 | 9.3041 | 89.01 | 4.3338 | 13.5427 | 59.3565 | 8.996 |
| Cu2 | 3.8477 | 7.7341 | 0.7589 | 9.0109 | 89.37 | 4.0874 | 19.5695 | 52.9266 | 8.637 |
| Cu3 | 4.8595 | 6.5958 | 1.1188 | 9.6731 | 93.26 | 3.6357 | 18.3259 | 60.5740 | 8.427 |
| Cm1 | 1.8363 | 5.2310 | 0.9426 | 4.2189 | 188.82 | 3.7090 | 9.5631 | 34.1659 | 9.001 |
| Cm2 | 1.7809 | 5.4673 | 1.0928 | 3.2382 | 205.36 | 4.3488 | 11.7666 | 30.3283 | 9.218 |
| Cm3 | 1.7465 | 5.2931 | 1.0932 | 3.2230 | 206.3 | 3.5491 | 9.9075 | 28.7071 | 9.281 |
| Cl1 | 1.1967 | 6.8215 | 1.2263 | 6.5282 | 239.72 | 4.2033 | 12.5989 | 19.9771 | 9.121 |
| Cl2 | 1.0787 | 6.8343 | 1.0694 | 5.0056 | 249.35 | 4.4219 | 11.6709 | 19.3922 | 9.166 |
| Cl3 | 1.2403 | 5.7126 | 1.2161 | 5.0521 | 288.73 | 3.5200 | 15.0941 | 22.0124 | 8.926 |
| Eu1 | 0.3321 | 1.1125 | 0.1838 | 2.0353 | 31.62 | 2.3708 | 3.0644 | 7.8339 | 9.277 |
| Eu2 | 0.3907 | 1.0258 | 0.2217 | 1.7563 | 33.96 | 3.9994 | 3.0867 | 8.4168 | 9.36 |
| Eu3 | 0.4490 | 1.2014 | 0.2772 | 1.7037 | 33.88 | 3.1414 | 2.8954 | 9.1488 | 9.042 |
| Em1 | 0.2598 | 0.3317 | 0.0314 | 1.0082 | 19.31 | 3.0687 | 0.8737 | 5.6989 | 9.45 |
| Em2 | 0.3767 | 0.4290 | 0.0631 | 1.1052 | 19 | 2.7487 | 0.9152 | 7.0178 | 9.252 |
| Em3 | 0.2736 | 0.3519 | 0.0482 | 0.9050 | 20.84 | 3.2288 | 0.9407 | 7.5054 | 9.167 |
| El1 | 0.3288 | 0.3170 | 0.0279 | 0.7150 | 15.43 | 3.6364 | 1.8112 | 5.2542 | 9.337 |
| El2 | 0.4283 | 0.4348 | 0.0259 | 0.8403 | 17.06 | 2.7631 | 2.2608 | 8.7224 | 9.414 |
| El3 | 0.3079 | 0.3575 | 0.0238 | 0.7231 | 15.42 | 3.0249 | 2.0759 | 7.6057 | 9.554 |
| Lu1 | 0.3079 | 0.6169 | 1.5970 | 3.0584 | 52.35 | 4.9747 | 1.6071 | 3.7184 | 9.207 |
| Lu2 | 0.2771 | 0.6295 | 1.3482 | 2.6298 | 50.34 | 4.1014 | 1.1607 | 2.1209 | 9.187 |
| Lu3 | 0.2495 | 0.6582 | 1.3854 | 2.9129 | 43.16 | 4.4944 | 1.3903 | 3.2053 | 9.211 |
| Lm1 | 0.1772 | 0.7001 | 1.1519 | 1.3175 | 31.19 | 4.2761 | 0.5740 | 1.7116 | 9.516 |
| Lm2 | 0.1909 | 0.6627 | 1.3887 | 1.5076 | 32.37 | 4.5379 | 0.7366 | 1.3772 | 9.282 |
| Lm3 | 0.1634 | 0.6047 | 1.1548 | 1.3175 | 33.82 | 4.1013 | 0.9598 | 1.5261 | 9.41 |
| Ll1 | 0.1462 | 0.5642 | 1.1587 | 0.9758 | 31.32 | 4.4074 | 1.6645 | 4.2102 | 9.533 |
| Ll2 | 0.1668 | 0.5918 | 1.1374 | 0.8363 | 32.88 | 7.0391 | 0.9790 | 3.3860 | 9.486 |
| Ll3 | 0.1600 | 0.5943 | 1.3337 | 0.7028 | 35.69 | 6.1229 | 1.3233 | 3.6117 | 9.495 |
| Wu1 | 0.7965 | 0.2495 | 0.7515 | 4.1301 | 53.63 | 4.4210 | 4.4005 | 15.6047 | 8.756 |
| Wu2 | 0.5523 | 0.3077 | 0.8569 | 3.7338 | 46.22 | 4.2909 | 4.3909 | 12.9549 | 9.104 |
| Wu3 | 0.8106 | 0.2475 | 0.8253 | 4.0836 | 64.5 | 4.5812 | 4.9522 | 16.5671 | 8.756 |
| Wm1 | 0.1739 | 0.1405 | 0.8436 | 1.1355 | 32.93 | 2.9817 | 2.2353 | 3.6755 | 9.479 |
| Wm2 | 0.2324 | 0.1501 | 0.8211 | 1.1760 | 35.34 | 3.3743 | 1.4477 | 4.8932 | 9.484 |
| Wm3 | 0.2598 | 0.1434 | 0.7592 | 1.0951 | 35.24 | 3.2726 | 1.9452 | 4.0116 | 9.518 |
| Wl1 | 0.8171 | 0.2665 | 0.6347 | 0.7311 | 28.31 | 3.3744 | 1.2819 | 11.0374 | 9.002 |
| Wl2 | 1.0513 | 0.2664 | 0.7699 | 0.7514 | 30.19 | 4.2761 | 1.2946 | 12.8034 | 8.942 |
| Wl3 | 0.8724 | 0.2214 | 0.9408 | 0.9071 | 38 | 3.8830 | 0.5389 | 10.8902 | 9 |
| Du1 | 1.8201 | 0.8955 | 0.8000 | 3.3444 | 61.78 | 8.1010 | 14.3495 | 42.3499 | 8.327 |
| Du2 | 1.5198 | 0.6280 | 0.7620 | 2.7984 | 58.7 | 5.9485 | 16.4828 | 34.1877 | 8.334 |
| Du3 | 1.2411 | 0.6456 | 0.7861 | 2.7428 | 54.81 | 7.2864 | 16.6040 | 33.6232 | 8.269 |
| Dm1 | 4.0169 | 1.5966 | 1.5548 | 4.1784 | 86.22 | 9.4100 | 38.5268 | 77.2176 | 8.219 |
| Dm2 | 4.2721 | 1.9441 | 1.3428 | 4.5222 | 82.48 | 6.2689 | 42.3852 | 77.6022 | 8.062 |
| Dm3 | 3.1437 | 1.5913 | 1.1354 | 4.1633 | 77.46 | 6.4430 | 48.5491 | 64.7652 | 8.109 |
| Dl1 | 3.3216 | 1.8042 | 2.2188 | 3.4556 | 113.4 | 5.9482 | 19.3941 | 54.3565 | 8.539 |
| Dl2 | 2.9654 | 1.2991 | 1.8385 | 3.3545 | 96.05 | 6.8213 | 19.3622 | 53.8403 | 8.259 |
| Dl3 | 3.7349 | 1.5565 | 1.9196 | 3.0512 | 99.3 | 7.6073 | 21.7793 | 59.5551 | 8.297 |
| Mu1 | 0.3322 | 0.3486 | 0.5005 | 2.0313 | 37.93 | 2.9092 | 6.5466 | 5.6279 | 9.337 |
| Mu2 | 0.2425 | 0.3824 | 0.5942 | 1.8230 | 38.55 | 2.1672 | 7.0536 | 3.9827 | 9.376 |
| Mu3 | 0.3425 | 0.4715 | 0.5934 | 1.6734 | 43.72 | 3.0545 | 7.9624 | 6.6559 | 9.076 |
| Mm1 | 0.2806 | 0.4601 | 0.4631 | 0.8221 | 37.43 | 4.1018 | 2.0855 | 2.4170 | 9.172 |
| Mm2 | 0.2322 | 0.3830 | 0.6290 | 0.9293 | 41.86 | 3.0251 | 1.6518 | 2.3426 | 9.304 |
| Mm3 | 0.3732 | 0.3817 | 0.6302 | 0.7817 | 38.44 | 3.7670 | 1.7921 | 3.3116 | 9.095 |
| Ml1 | 0.3978 | 0.4083 | 1.0130 | 0.2782 | 60.33 | 2.5307 | 3.3833 | 6.1354 | 8.989 |
| Ml2 | 0.2806 | 0.3745 | 0.7956 | 0.2904 | 54.89 | 2.9817 | 4.1964 | 5.1027 | 9.186 |
| Ml3 | 0.3220 | 0.3838 | 0.9469 | 0.3166 | 54.51 | 3.3450 | 4.0051 | 4.6154 | 9.108 |

**Table s2 Sequencing data and OTU statistics of bacterial samples**

| **Sample** | **Raw reads** | **Clean reads** | **Effective reads** | **GC(%)** | OTUs |
| --- | --- | --- | --- | --- | --- |
| Au1 | 79508 | 79430 | 78396 | 56.6 | 4565 |
| Au2 | 74788 | 74708 | 73834 | 56.9 | 4556 |
| Au3 | 88644 | 88559 | 87396 | 56.7 | 4802 |
| Am1 | 86810 | 86712 | 85620 | 56.8 | 4444 |
| Am2 | 84304 | 84205 | 83165 | 56.6 | 4892 |
| Am3 | 91063 | 90968 | 89909 | 56.7 | 4869 |
| Al1 | 88766 | 88659 | 87685 | 56.6 | 4479 |
| Al2 | 90726 | 90614 | 89416 | 56.8 | 4656 |
| Al3 | 85743 | 85656 | 84691 | 56.6 | 4300 |
| Cu1 | 77849 | 77779 | 76846 | 56.1 | 4481 |
| Cu2 | 79011 | 78933 | 78037 | 55.9 | 4573 |
| Cu3 | 91111 | 91035 | 89946 | 55.7 | 4949 |
| Cm1 | 88443 | 88381 | 87330 | 56.4 | 4665 |
| Cm2 | 85649 | 85562 | 84535 | 56.3 | 4666 |
| Cm3 | 86691 | 86599 | 85731 | 56.2 | 4697 |
| Cl1 | 88962 | 88858 | 87664 | 56.5 | 4683 |
| Cl2 | 87051 | 86956 | 85840 | 56.6 | 4739 |
| Cl3 | 89918 | 89819 | 88642 | 56.7 | 4905 |
| Eu1 | 85586 | 85500 | 84476 | 55.9 | 4673 |
| Eu2 | 59659 | 59594 | 58614 | 56 | 4120 |
| Eu3 | 86478 | 86402 | 85426 | 55.7 | 4587 |
| Em1 | 85684 | 85582 | 84645 | 56.2 | 4887 |
| Em2 | 90084 | 89992 | 88840 | 56.2 | 4757 |
| Em3 | 92299 | 92197 | 91054 | 56.1 | 4775 |
| El1 | 62556 | 62504 | 61780 | 55.9 | 4354 |
| El2 | 88178 | 88103 | 87050 | 56 | 4849 |
| El3 | 85550 | 85480 | 84444 | 56.2 | 5081 |
| Wu1 | 89278 | 89185 | 87849 | 55.2 | 4126 |
| Wu2 | 81151 | 81051 | 79942 | 55.3 | 4343 |
| Wu3 | 89805 | 89704 | 88333 | 55.4 | 4501 |
| Wm1 | 91093 | 90988 | 89671 | 55.9 | 4132 |
| Wm2 | 87634 | 87531 | 86209 | 56.5 | 3995 |
| Wm3 | 92192 | 92095 | 90944 | 56.1 | 4142 |
| Wl1 | 88714 | 88617 | 87284 | 56.1 | 4572 |
| Wl2 | 85077 | 84965 | 83803 | 56.2 | 4358 |
| Wl3 | 85790 | 85699 | 84528 | 56.2 | 4513 |
| Lu1 | 92376 | 92282 | 91106 | 55.5 | 3602 |
| Lu2 | 91364 | 91265 | 89801 | 55.7 | 3720 |
| Lu3 | 86622 | 86527 | 85402 | 55.9 | 3478 |
| Lm1 | 86761 | 86688 | 85456 | 56.2 | 3874 |
| Lm2 | 85555 | 85468 | 84357 | 56.3 | 3901 |
| Lm3 | 91968 | 91845 | 90389 | 55.8 | 3589 |
| Ll1 | 89646 | 89556 | 88337 | 56 | 4040 |
| Ll2 | 77126 | 77038 | 76028 | 56.1 | 4072 |
| Ll3 | 63187 | 63126 | 62376 | 56.1 | 3770 |
| Du1 | 62186 | 62111 | 61361 | 55.9 | 3839 |
| Du2 | 88171 | 88081 | 86947 | 55.9 | 4578 |
| Du3 | 85988 | 85876 | 84760 | 55.9 | 4544 |
| Dm1 | 92331 | 92203 | 90740 | 56.6 | 5225 |
| Dm2 | 84920 | 84820 | 83647 | 57.2 | 3925 |
| Dm3 | 85396 | 85290 | 84095 | 56.8 | 4287 |
| Dl1 | 87820 | 87702 | 86465 | 56.9 | 5242 |
| Dl2 | 90909 | 90778 | 89501 | 57 | 5201 |
| Dl3 | 92167 | 92039 | 90720 | 57.2 | 4995 |
| Mu1 | 84098 | 84031 | 82822 | 55.2 | 4536 |
| Mu2 | 87221 | 87128 | 85943 | 55 | 4710 |
| Mu3 | 66146 | 66075 | 65278 | 55 | 4360 |
| Mm1 | 87171 | 87077 | 85923 | 55.9 | 5384 |
| Mm2 | 89838 | 89735 | 88496 | 55.9 | 5402 |
| Mm3 | 71505 | 71417 | 70514 | 55.9 | 4941 |
| Ml1 | 85734 | 85642 | 84422 | 56.1 | 5179 |
| Ml2 | 84915 | 84814 | 83787 | 55.8 | 4747 |
| Ml3 | 85558 | 85466 | 84277 | 56.6 | 5107 |

**Table s3 Sequencing data and OTU statistics of fungal samples**

| **Sample** | **Raw reads** | **Clean reads** | **Effective reads** | **GC(%)** | **OTUs** |
| --- | --- | --- | --- | --- | --- |
| Au1 | 90738 | 90637 | 88315 | 47.1 | 372 |
| Au2 | 84776 | 84675 | 82130 | 46.5 | 409 |
| Au3 | 89129 | 89010 | 86429 | 47.1 | 365 |
| Am1 | 86585 | 86464 | 83867 | 46.2 | 376 |
| Am2 | 91614 | 91463 | 88438 | 47.5 | 433 |
| Am3 | 85867 | 85778 | 81030 | 51.1 | 388 |
| Al1 | 89823 | 89694 | 87505 | 47.5 | 366 |
| Al2 | 88335 | 88234 | 85660 | 46.4 | 384 |
| Al3 | 85939 | 85857 | 83718 | 46.1 | 354 |
| Cu1 | 89158 | 89074 | 82061 | 48.5 | 378 |
| Cu2 | 84336 | 84266 | 81187 | 47.4 | 398 |
| Cu3 | 84920 | 84856 | 79994 | 48.1 | 400 |
| Cm1 | 90699 | 90440 | 86987 | 46.8 | 373 |
| Cm2 | 86293 | 86177 | 82976 | 47.1 | 360 |
| Cm3 | 92121 | 91961 | 89227 | 47.6 | 381 |
| Cl1 | 87139 | 86789 | 83662 | 46.8 | 379 |
| Cl2 | 88828 | 88728 | 85704 | 47 | 356 |
| Cl3 | 86056 | 85782 | 82091 | 46.6 | 354 |
| Eu1 | 89818 | 89724 | 87242 | 49.6 | 324 |
| Eu2 | 84944 | 84874 | 82772 | 49.7 | 314 |
| Eu3 | 74281 | 74223 | 72088 | 50.8 | 298 |
| Em1 | 86240 | 86153 | 83938 | 47 | 363 |
| Em2 | 84329 | 84149 | 81978 | 47.7 | 364 |
| Em3 | 91104 | 91035 | 89178 | 48.2 | 377 |
| El1 | 92109 | 92032 | 90004 | 47.1 | 352 |
| El2 | 84399 | 84324 | 82509 | 46.9 | 438 |
| El3 | 85983 | 85906 | 83812 | 47.2 | 358 |
| Lu1 | 88176 | 88106 | 86248 | 46.8 | 332 |
| Lu2 | 89162 | 89085 | 86676 | 47.5 | 344 |
| Lu3 | 84517 | 84469 | 82382 | 47.5 | 338 |
| Lm1 | 91139 | 91029 | 88995 | 45.2 | 359 |
| Lm2 | 87540 | 87457 | 85887 | 45.1 | 367 |
| Lm3 | 92355 | 92260 | 90142 | 46.7 | 346 |
| Ll1 | 85762 | 85702 | 83229 | 44 | 274 |
| Ll2 | 87033 | 86955 | 84082 | 44.5 | 507 |
| Ll3 | 84147 | 84005 | 80304 | 45.1 | 638 |
| Wu1 | 89285 | 89210 | 85390 | 48.6 | 311 |
| Wu2 | 88787 | 88722 | 84883 | 50.8 | 551 |
| Wu3 | 57735 | 57683 | 54818 | 49.9 | 524 |
| Wm1 | 91777 | 91663 | 88994 | 45.3 | 523 |
| Wm2 | 91078 | 91000 | 88857 | 45.7 | 356 |
| Wm3 | 88104 | 88022 | 85979 | 46.5 | 523 |
| Wl1 | 86376 | 86256 | 82549 | 46.3 | 513 |
| Wl2 | 89200 | 89128 | 85663 | 46.8 | 605 |
| Wl3 | 89685 | 89592 | 86183 | 45.8 | 365 |
| Du1 | 86344 | 86265 | 84693 | 48.2 | 318 |
| Du2 | 50806 | 50741 | 49523 | 44 | 283 |
| Du3 | 85167 | 85094 | 82788 | 46.1 | 321 |
| Dm1 | 84541 | 84454 | 81818 | 45.5 | 485 |
| Dm2 | 91676 | 91597 | 87750 | 45.7 | 406 |
| Dm3 | 87288 | 86825 | 83165 | 46.8 | 480 |
| Dl1 | 84828 | 84758 | 81996 | 48.3 | 627 |
| Dl2 | 92073 | 91987 | 88576 | 48.1 | 605 |
| Dl3 | 86128 | 86039 | 82187 | 47.5 | 594 |
| Mu1 | 85968 | 85911 | 84091 | 51 | 308 |
| Mu2 | 84163 | 84093 | 82405 | 50.5 | 319 |
| Mu3 | 90261 | 90185 | 88318 | 51 | 345 |
| Mm3 | 85639 | 85561 | 83481 | 52.1 | 298 |
| Mm2 | 84389 | 84313 | 82107 | 51 | 323 |
| Mm1 | 84707 | 84648 | 82859 | 51.3 | 337 |
| Ml1 | 90300 | 90223 | 88499 | 47.1 | 420 |
| Ml2 | 87823 | 87750 | 86017 | 46.8 | 537 |
| Ml3 | 88916 | 88854 | 87053 | 47.2 | 525 |

Table s4 Statistics of relative abundance distribution of species at phylum level

Bacterium

| **Phylum** | **A** | **C** | **E** | **W** | **L** | **D** | **M** |
| --- | --- | --- | --- | --- | --- | --- | --- |
| Proteobacteria | 0.3065 | 0.3338 | 0.4777 | 0.4122 | 0.4979 | 0.2991 | 0.3980 |
| Planctomycetota | 0.1916 | 0.1559 | 0.1094 | 0.1385 | 0.0771 | 0.1392 | 0.1106 |
| Acidobacteriota | 0.1521 | 0.1181 | 0.0658 | 0.1025 | 0.0965 | 0.2032 | 0.0755 |
| Crenarchaeota | 0.0662 | 0.1182 | 0.0553 | 0.0957 | 0.0687 | 0.0549 | 0.0675 |
| Actinobacteriota | 0.0554 | 0.0552 | 0.0709 | 0.0494 | 0.0682 | 0.0412 | 0.0511 |
| Chloroflexi | 0.0295 | 0.0350 | 0.0339 | 0.0284 | 0.0408 | 0.0460 | 0.0401 |
| Bacteroidota | 0.0260 | 0.0354 | 0.0393 | 0.0460 | 0.0224 | 0.0298 | 0.0498 |
| (Unassigned) | 0.0379 | 0.0299 | 0.0257 | 0.0231 | 0.0261 | 0.0300 | 0.0345 |
| Verrucomicrobiota | 0.0323 | 0.0293 | 0.0147 | 0.0237 | 0.0169 | 0.0221 | 0.0345 |
| Gemmatimonadota | 0.0280 | 0.0231 | 0.0196 | 0.0227 | 0.0141 | 0.0309 | 0.0253 |
| Myxococcota | 0.0180 | 0.0129 | 0.0145 | 0.0090 | 0.0119 | 0.0386 | 0.0265 |
| others | 0.0565 | 0.0530 | 0.0732 | 0.0487 | 0.0593 | 0.0647 | 0.0864 |

Fungi

| **Phylum** | **A** | **C** | **E** | **W** | **L** | **D** | **M** |
| --- | --- | --- | --- | --- | --- | --- | --- |
| Ascomycota | 0.5251 | 0.6694 | 0.7887 | 0.6946 | 0.4982 | 0.8148 | 0.7493 |
| (Unassigned) | 0.2302 | 0.1366 | 0.1333 | 0.1305 | 0.0627 | 0.0864 | 0.1538 |
| Basidiomycota | 0.0456 | 0.0896 | 0.0408 | 0.0324 | 0.2754 | 0.0394 | 0.0879 |
| Mortierellomycota | 0.1628 | 0.0695 | 0.0100 | 0.1060 | 0.1194 | 0.0340 | 0.0040 |
| Glomeromycota | 0.0273 | 0.0163 | 0.0133 | 0.0287 | 0.0333 | 0.0007 | 0.0031 |
| others | 0.0089 | 0.0185 | 0.0138 | 0.0077 | 0.0109 | 0.0247 | 0.0017 |

Table s5

Bacterium

| **Sampels** | **Unassigned** | **Stenotrophomonas** | **Ralstonia** | **Acinetobacter** | **Herbaspirillum** | **Delftia** | **Pirellula** | **MND1** | **Pir4_lineage** | **Bacillus** | **Nitrospira** | **Phyllobacterium** | **Sphingomonas** | **RB41** | **Gemmata** | **Povalibacter** | **Bryobacter** | **Ohtaekwangia** | **Subgroup_10** | **Stenotrophobacter** |
| --- | --- | --- | --- | --- | --- | --- | --- | --- | --- | --- | --- | --- | --- | --- | --- | --- | --- | --- | --- | --- |
| **TN** | 3.61E-07 | 5.02E-05 | 0.000243058 | 1.01E-14 | 6.60E-06 | 6.36E-06 | 1.72E-08 | 0.483672269 | 3.12E-05 | 0.609891264 | 0.155103128 | 8.40E-11 | 0.021220055 | 0.000810513 | 3.85E-05 | 3.00E-07 | 8.53E-16 | 0.000257285 | 0.191763815 | 0.079351646 |
| **TP** | 0.000150718 | 0.006134197 | 0.037192741 | 2.97E-05 | 0.021808706 | 0.006134197 | 0.001879211 | 0.009558982 | 0.01392268 | 0.283873432 | 0.85776978 | 0.000130339 | 0.003572477 | 2.45E-09 | 0.005184444 | 0.047651693 | 0 | 0.248400578 | 0.607750268 | 0.693880011 |
| **TK** | 0.000384484 | 0.005152156 | 0.124092892 | 8.28E-05 | 0.031612552 | 0.001408795 | 0.000528414 | 0.040088218 | 0.521760517 | 0.008892449 | 0.299806742 | 0.00384654 | 0.23185669 | 0.000969881 | 0.000130799 | 0.353591419 | 0.000132419 | 0.530346049 | 0.290730457 | 0.009536818 |
| **AP** | 0.001345412 | 0.008821243 | 0.045773424 | 7.78E-08 | 0.009969888 | 0.001948846 | 2.61E-09 | 8.18E-05 | 0.004831209 | 0.051309777 | 0.011468913 | 1.46E-05 | 0.285410431 | 4.90E-06 | 7.26E-06 | 2.28E-05 | 9.27E-13 | 3.51E-05 | 0.10952207 | 0.031718346 |
| **AK** | 6.63E-07 | 2.57E-07 | 5.44E-06 | 7.29E-06 | 3.59E-06 | 2.82E-07 | 4.04E-05 | 0.274455523 | 0.154803772 | 0.796200314 | 0.807208685 | 4.77E-06 | 0.839279604 | 8.03E-06 | 0.005825705 | 0.00788421 | 2.65E-07 | 0.004485466 | 0.12029904 | 0.08304736 |
| **NH4+** | 0.522694325 | 0.839742123 | 0.549136458 | 0.016358404 | 0.798796292 | 0.451777961 | 0.018505272 | 0.139546209 | 0.017304526 | 1.29E-05 | 0.316331191 | 0.111328186 | 0.138062041 | 0.047524974 | 0.001096962 | 0.388172639 | 0.000619296 | 0.728694576 | 0.535094141 | 0.045901323 |
| **NO3** | 0.008861256 | 0.002513537 | 0.000583941 | 0.011329247 | 0.000642752 | 0.001429107 | 0.009919782 | 0.827876123 | 0.47710831 | 0.584066038 | 0.51834606 | 0.000333352 | 0.351245722 | 0.071134752 | 0.491918219 | 0.032427043 | 0.001972821 | 0.069161887 | 0.045218082 | 0.014390921 |
| **SOC** | 3.86E-06 | 0.000697248 | 0.001545623 | 0 | 2.12E-05 | 8.93E-05 | 4.71E-09 | 0.554502355 | 4.25E-05 | 0.500488446 | 0.091809147 | 0 | 0.023012773 | 0.002348727 | 1.59E-05 | 2.77E-07 | 0 | 0.001255451 | 0.41578801 | 0.040239677 |
| **PH** | 7.76E-06 | 1.20E-05 | 8.55E-05 | 1.44E-08 | 5.73E-06 | 3.08E-06 | 2.27E-06 | 0.507780239 | 0.002178194 | 0.08320612 | 0.062574161 | 8.62E-08 | 0.262955349 | 0.01509087 | 0.000593971 | 0.000296946 | 3.73E-10 | 0.005766707 | 0.381769187 | 0.299453752 |

Fungi

| **Samples** | **(Unassigned)** | **Fusarium** | **Mortierella** | **Aspergillus** | **Rhizoctonia** | **Lecanicillium** | **Acrocalymma** | **Rhodotorula** | **Preussia** | **Metacordyceps** | **Plectosphaerella** | **Stachybotrys** | **Trichosporon** | **Microascus** | **Lasionectria** | **Acremonium** | **Nectria** | **Phaeophleospora** | **Botryosphaeria** | **Verticillium** |
| --- | --- | --- | --- | --- | --- | --- | --- | --- | --- | --- | --- | --- | --- | --- | --- | --- | --- | --- | --- | --- |
| **TN** | 0.000443286 | 0.023354462 | 0.018295593 | 0.102884387 | 0.054053183 | 0.623452828 | 0.474919349 | 0.000205842 | 4.73E-07 | 8.55E-07 | 0.593198465 | 0.969618976 | 0.131058817 | 0.310511007 | 0.006698667 | 0.260784053 | 0.595918675 | 4.27E-05 | 3.84E-05 | 0.096114366 |
| **TP** | 0.000975655 | 0.301219375 | 1.06E-05 | 0.649026723 | 2.56E-05 | 0.288392022 | 0.335097873 | 0.006386608 | 1.01E-05 | 1.98E-08 | 0.022288332 | 0.040391612 | 0.814471673 | 0.049580647 | 0.000208414 | 0.001045872 | 0.058256966 | 0.000516802 | 4.21E-05 | 0.023077978 |
| **TK** | 0.00317926 | 0.66748449 | 1.41E-08 | 0.109536035 | 0.000119273 | 0.202541847 | 0.081620175 | 0.000575824 | 0.276455799 | 0.032706516 | 0.083115813 | 0.002190671 | 0.137942751 | 0.002673805 | 0.016653133 | 0.705545997 | 0.000759393 | 0.197122933 | 0.128133464 | 0.529452649 |
| **AP** | 0.000329884 | 0.189185781 | 0.000239973 | 0.38116547 | 0.00010139 | 0.282663113 | 0.13003732 | 3.56E-06 | 2.33E-06 | 1.19E-06 | 0.357834405 | 0.148649812 | 0.095260252 | 0.327775767 | 0.000169685 | 0.347666613 | 0.009657451 | 0.001640271 | 0.000562984 | 0.111624775 |
| **AK** | 1.20E-06 | 0.007165101 | 0.043450988 | 0.022753451 | 0.096906644 | 0.257888686 | 0.01677848 | 0.001470526 | 3.96E-06 | 0.022154581 | 0.077649848 | 0.895563559 | 0.050010437 | 0.451890994 | 0.054042862 | 0.491681265 | 0.03497594 | 0.000333727 | 0.011827901 | 0.291686091 |
| **NH4+** | 9.72E-06 | 7.73E-05 | 0.002171142 | 0.357439995 | 0.069507909 | 0.470963439 | 0.000368131 | 0.018326678 | 0.125233864 | 0.788754954 | 0.06503561 | 0.188415649 | 0.969321633 | 0.021889325 | 0.079310629 | 0.216872219 | 0.006124687 | 0.032735244 | 0.062632018 | 0.483748523 |
| **NO3** | 2.74E-05 | 0.001351355 | 0.406777596 | 0.000298192 | 0.022815982 | 0.158004043 | 0.219642456 | 0.151728165 | 1.16E-06 | 0.690340182 | 0.064450082 | 0.026888833 | 0.372502927 | 0.20527059 | 0.867247475 | 0.029040004 | 0.691656228 | 0.19531251 | 0.944607751 | 0.025358708 |
| **soc** | 0.001378815 | 0.008296316 | 0.010519398 | 0.246540547 | 0.105229034 | 0.872130498 | 0.713081801 | 0.000309685 | 1.63E-07 | 2.87E-07 | 0.29857425 | 0.999850598 | 0.380851217 | 0.59177796 | 0.002000111 | 0.374528762 | 0.766218487 | 6.84E-05 | 4.74E-05 | 0.024612087 |
| **PH** | 0.000327549 | 0.042065386 | 0.128331159 | 0.074678759 | 0.149660067 | 0.076873461 | 0.26077827 | 0.003094581 | 0.001047307 | 0.003317084 | 0.480506132 | 0.596698169 | 0.088687878 | 0.354865388 | 0.119832034 | 0.842367599 | 0.356732982 | 0.014917998 | 0.067910429 | 0.966444495 |
